# Possible Time-Dependent Effect of Ions and Hydrophilic Surfaces on the Electrical Conductivity of Aqueous Solutions

**DOI:** 10.3390/ijms13044048

**Published:** 2012-03-27

**Authors:** Nada Verdel, Igor Jerman, Rok Krasovec, Peter Bukovec, Marija Zupancic

**Affiliations:** 1 Institute Bion d. o. o., Stegne 21, 1000 Ljubljana, Slovenia; E-Mails: igor.jerman@bion.si (I.J.); rok.krasovec@bion.si (R.K.); 2 Department of Chemistry, University of Ljubljana, Aškerčeva 5, 1000 Ljubljana, Slovenia; E-Mails: peter.bukovec@fkkt.uni-lj.si (P.B.); marija.zupancic@fkkt.uni-lj.si (M.Z.)

**Keywords:** autothixotropy, conductivity, exclusion zone, extremely dilute solutions, water

## Abstract

The purpose of this work was to determine the influence of mechanical and electrical treatment on the electrical conductivity of aqueous solutions. Solutions were treated mechanically by iteration of two steps: 1:100 dilution and vigorous shaking. These two processes were repeated until extremely dilute solutions were obtained. For electrical treatment the solutions were exposed to strong electrical impulses. Effects of mechanical (as well as electrical) treatment could not be demonstrated using electrical conductivity measurements. However, significantly higher conductivity than those of the freshly prepared chemically analogous solutions was found in all aged solutions except for those samples stored frozen. The results surprisingly resemble a previously observed weak gel-like behavior in water stored in closed flasks. We suggest that ions and contact with hydrophilic glass surfaces could be the determinative conditions for the occurrence of this phenomenon.

## 1. Introduction

It is generally thought that the impact of surfaces on the continuous phase of bulk water extends to a distance of no more than a few water molecule layers. Pollack and coworkers [1,2] on the other hand, report that colloidal and molecular solutes suspended in aqueous solutions are extensively excluded from the vicinity of various hydrophilic surfaces. The depth of the solute-free zone (exclusion zone or EZ) is typically several hundred microns. NMR and IR images indicate that EZ water has lower mobility and is more ordered than bulk water [2,3]. According to Guckenberger and coworkers [4], the thin water layer next to a hydrophilic surface exhibits a surprisingly increased conductivity, higher than that of bulk water by up to five orders of magnitude. They ascribed the increased conductivity to a proton hopping mechanism along water structured at the surfaces. Similarly, Sasaki [5] found that conductivity of collagen, the most abundant protein in mammals, depends remarkably on the amount of hydration water.

In our study we continued the work of Elia and coworkers [6]. Elia and coworkers explored the physico-chemical properties of aqueous solutions of NaHCO_3_ treated mechanically by iterated dilution and succussion (vigorous shaking). They repeated the processes to extreme dilution, where the chemical composition of the end solution was identical to that of the solvent. They measured electrical conductivities of aged, extremely dilute solutions, and compared the results with electrical conductivity values of one-day-old untreated analogous solutions [6–12]. They noticed significantly higher electrical conductivities than in untreated solutions. They attributed their findings to the ordering of water triggered by the input of kinetic energy during mechanical treatment (succussion). Namely, the high mobility of protons under a gradient of electrical potential may be explained by the Grotthuss mechanism, for which the structuring (or ordering) of water molecules implies a faster proton conductivity [13–17].

However, they found excess conductivities only when solutions were left to stand undisturbed for some time. Which is surprisingly similar to the observations of Vybíral, who noticed that distilled water, left to stand undisturbed for some time, develops “autothixotropic” properties, where ions seem to play an important role [18,19]; thixotropy is a property of some gels or liquids that under normal conditions are highly viscous whereas during mechanical processing their viscosity diminishes.

With combining the insights of Pollack [2], Elia [6] and those of Vybíral and Voráček [18,19], the following working hypothesis was developed: When water is left to stand undisturbed, its properties change to “autothixotropic” [19], which plays a major role in the proton conducting properties of water detected by conductivity measurements of aged sodium hydrogen carbonate solutions by Elia and coworkers [6]; on the other hand, previous mechanical or electrical treatment has no influence on the conductivity values of aged solutions. Due to higher ratios between glass contact surface and volume, we expect higher values of excess conductivity in smaller volumes. The same would be expected from exposure to light. Whereas due to the reduced translational mobility of water molecules, thixotropic properties cannot be established if solutions are aged frozen.

We tested our working hypothesis by measuring the electrical conductivity of aqueous solutions of sodium hydrogen carbonate as a function of time, volume and ageing condition, mechanical and electrical treatment and temperature of the conductivity measurements. In electrical treatment, solutions were exposed to a high voltage pulsed electric field [20]. Untreated solutions were aged and measured in the same way as the sample solutions.

## 2. Results and Discussion

This study was performed in order to better understand the phenomenon of change in the physico-chemical properties of mechanically treated extremely dilute solutions, as reported by Eliàs’ research group [6–12]. They attributed the changed properties to the input of kinetic energy during mechanical treatment, whereby dissipative structures are supposed to occur due to self-organizing abilities of water.

According to our hypothesis, the cause for excess conductivities in aged aqueous solutions are the autothixotropic properties of water that develop spontaneously when water is left to stand undisturbed for some time. We tested our hypothesis by measuring the conductivity of fresh, one day old, NaHCO_3_ solutions. We found that on the first day after treatment the conductivity values of solutions subjected to mechanical or electrical treatment did not differ from the chemically analogous untreated solutions. Therefore, we aged the treated as well as the untreated solutions. Solutions were kept in three different volumes with different ratios of the contact surface (glass) and the liquid. Furthermore, we aged the solutions exposed to light (condition PR), protected from light (ST) and at low temperatures (MD).

The electrical conductivity measurements of mechanically and electrically treated solutions and untreated controls (CON) proceeded in combination with chemical analyses by ICP-MS. No excess conductivity of the treated samples was found immediately after their preparation, whereas in all aged (310 or 370 days old) solutions higher conductivity values were obtained than in chemically analogous one-day-old untreated solutions. However, we found no differences in conductivity values between aged mechanically (or electrically) treated solutions and aged untreated solutions, and no excess conductivity was found in frozen samples. Therefore, we propose time-related changes in the structure of water, where ions and hydrophilic surfaces seem to play an important role [21].

### 2.1. Repeatability of Conductivity Measurements and Influence of Treatment

**Figure 1:**
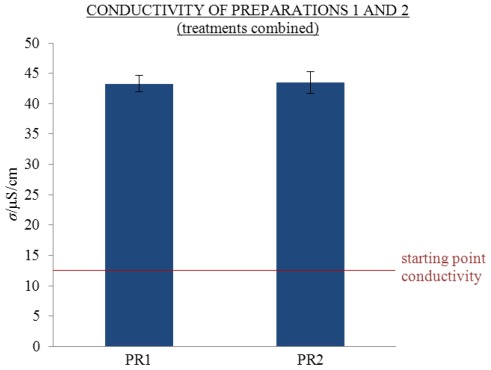
Repeatability of conductivity values on ageing of preparations PR1 and PR2. Average *σ* at the start (starting point conductivity) and 310th day with standard error (SE) intervals, measured at 25 °C and 1000 Hz when treatments are combined; PR1 (29 replicate solutions), PR2 (27 replicate solutions).

**Figure 2:**
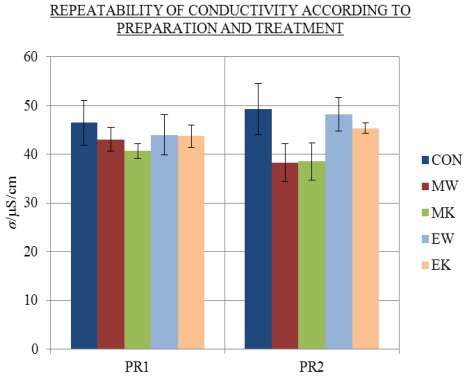
Repeatability of average *σ* with standard error (SE) intervals at 25 °C and 1000 Hz after 310 days of ageing of 2 mL treatments CON (blue), MW (red), MK (green), EW (light blue) and EK (orange) of preparations PR1 and PR2; number of replicates, N (/), of PR1: CON (5), MW (7), MK (7), EW (5), EK (5); number of replicates of PR2: CON (5), MW (5), MK (7), EW (5), EK (5).

Before measuring the potential excess conductivity values of the samples, the repeatability of the conductivity values of 2 mL solutions aged in 2.5 mL flasks was tested. Two preparations, PR1 and PR2, of samples were measured at 25 °C after 310 days (see Figure 1 with combined treatments). The measured conductivity values (*σ*) of aged treatments CON, MW, MK, EW and EK of preparations PR1 and PR2 were compared (see Figure 2 and the supplementary information).

The repeatability of the conductivity values was compared using a linear mixed model (LMM). The variation of conductivity at 1000 Hz between the two preparations was insignificant (*p* = 0.887) (see Figure 1 and the supplementary information). Therefore we concluded that conductivity values of solutions aged for 310 days at 1000 Hz are repeatable.

Figure 2 shows that treatments had no significant effect on the conductivity values of aged solutions (see the supplementary information). The conductivity of aged control solutions (CON) was similar to the values of the aged treated samples (MW, MK, EW and EK). In contrast to Elia and Niccoli’s [7] findings, mechanical treatment by iterated dilution and succussion (as well as treatment with strong electrical impulses) had no essential influence on the conductivity. Rather small differences were disregarded due to different conductivity values at 1000 Hz of treatments in different preparations (see Figure 2).

Our results are similar to the observations of Holandino and coworkers [22]. They compared the time evolution of the conductivity of distilled water treated by iterated dilution and mechanical shaking, starting with a solution of vynchristine sulphate, to the conductivity of untreated distilled water. They found that the electrical conductivity develops with time equally in both, the treated and untreated samples. In contrast, Holysz and coworkers [23] found that magnetic fields significantly influence the electrical conductivity of electrolytic solutions.

### 2.2. Influence of Ageing Volume

**Figure 3:**
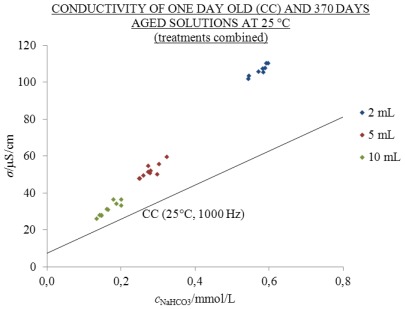
*σ* at 25 °C and 1000 Hz as a function of *c*_NaHCO3_ of one-day-old (CC) and 370 days in 2 mL (blue), 5 mL (red) and 10 mL (green) volumes of 20 mL flasks under condition PR aged solutions; points in the graph represent individual measurements; number of replicate solutions, N (/): 2 mL (8), 5 mL (10), 10 mL (10). Treatments are combined; conductivity above the CC line is excess.

The influence of the ageing volume on conductivity values was tested with ageing in three different volumes of 2, 5 and 10 mL in 20 mL flasks under the condition PR (exposed to daylight) for 370 days (see Figure 3 and the supplementary information).

If we draw a line from an arbitrary point on Figure 3 parallel with the abscissa to the calibration curve (CC), we can read the concentration of a one-day-old solution with the same conductivity. In this way, 2, 5 and 10 mL volumes of aged solutions have the same conductivity as one-day-old solutions with more than twice, 1.9- and 1.5-times higher concentrations of NaHCO_3_, respectively. The ratio of the excess and measured conductivity, *σ*^E^/*σ*, in 2, 5 and 10 mL volumes amounts to 0.43, 0.36 and 0.27, respectively. This means that for 2, 5 and 10 mL volumes 45.9, 18.6 and 8.3 μS/cm of excess conductivity was found.

**Figure 4:**
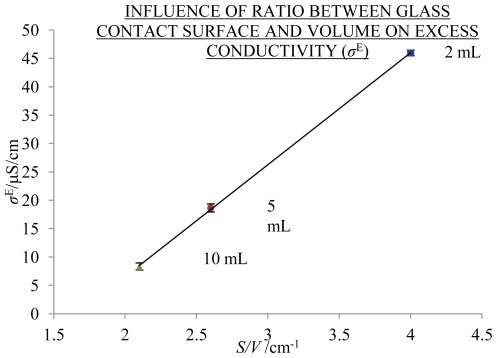
Influence of the ratio of the glass contact surface and the volume, *S/V*, on average *σ*^E^ with SE intervals, measured at 25 °C and 1000 Hz, aged for 370 days in 2 mL (blue), 5 mL (red) and 10 mL (green) volumes of 20-mL flasks; number of replicate solutions, N (/): 2 mL (8), 5 mL (10), 10 mL (10). The abscissa starts at 1.5 cm^−1^.

**Figure 5:**
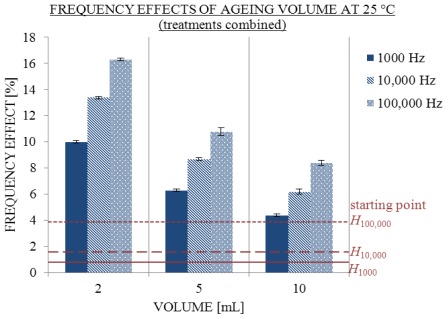
Influence of ageing volume on average frequency effects with SE intervals at 1000 (filled), 10,000 (striped) and 100,000 Hz (dotted) 25 °C. Conductivity measured at start (starting point H) and 370th day in 2, 5 and 10 mL; number of replicate solutions, N (/): 2 mL (8), 5 mL (10), 10 mL (10).

Different volumes had different ratios of the contact surface of the liquid with glass and the volume (*S/V*). At higher values of *S/V*, a larger share of the solution was within the border zone near the hydrophilic surface, or EZ, and therefore a higher *σ*^E^ may be expected (see Figure 4); *S/V* for 2, 5 and 10 mL was 4.0 cm^−1^, 2.6 cm^−1^ and 2.1 cm^−1^, respectively. In Figure 5 influence of ageing volume on frequency effects is presented.

The *σ*^E^ values after ageing for 370 days in 2, 5 and 10 mL cover 43.3, 36.2 and 26.9% of the measured conductivity, respectively. This means that in 2, 5 and 10 mL solutions 46.0, 18.6 and 8.3 μS/cm of excess conductivity was found, respectively (see Figure 3).

The ratio of the glass contact surface and volume (*S/V*) tells us the proportion of border zone water that could, according to Pollack and coworkers [2], potentially influence the ordering of water. With increasing ratio *S/V*, *σ*^E^ values increase via the following equation (see Figure 4):

(1) $$\sigma^{\text{E}}\;[\mu \text{S}/\text{cm}]=20\times S/V\;[{\text{cm}}^{-1}]-33$$

where *R*^2^ = 0.999. From Equation (1) we may deduce that hydrophilic surfaces could be a factor influencing the increase of conductivity.

Furthermore, the same trend as in Equation (1) can be noticed for frequency effects (see Figure 5). After 370 days the frequency effects of 2, 5 and 10 mL solutions were all higher than on the first day after treatment. The frequency effects observed for 2 mL solutions were significantly higher than those for 5 and 10 mL. Furthermore, all frequency effects of aged solutions were significantly higher than those of one-day-old solutions with the same concentration of NaHCO_3_. Correlations between the frequency effects of aged and one day old (fresh) solutions with an equal concentration of sodium are described in the Equations (2), (3) and (4). Again, the same order can be noticed:

(2) $$V=2\;\text{mL}:\;H_{AGED}=1.9\times H_{FRESH}+6.2;\;R^{2}=0.9652$$

(3) $$V=5\;\text{mL}:\;H_{AGED}=1.5\times H_{FRESH}+4.7;\;R^{2}=0.9287$$

(4) $$V=10\;\text{mL}:\;H_{AGED}=1.3\times H_{FRESH}+3.2;\;R^{2}=0.9563$$

### 2.3. Influence of Ageing Condition and Temperature

**Figure 6:**
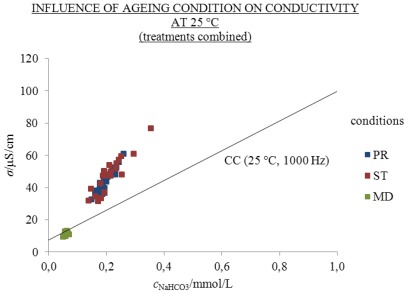
*σ* at 25 °C and 1000 Hz as a function of *c*_NaHCO3_ of one day old (CC) and 310 days in 2 mL volume of 2.5 mL flasks under conditions PR (blue), ST (red) and MD (green) aged solutions; points in the graph represent individual measurements; number of replicate solutions, N (/): PR (28), ST (20), MD (20). Treatments are combined; conductivity above the CC line is excess.

**Figure 7:**
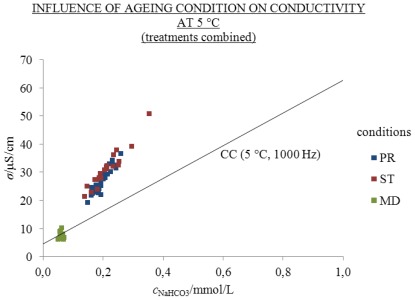
*σ* at 5 °C and 1000 Hz as a function of *c*_NaHCO3_ of one day old (CC) and 310 days in 2 mL volume of 2.5 mL flasks under conditions PR (blue), ST (red) and MD (green) aged solutions; points in the graph represent individual measurements; number of replicate solutions, N (/): PR (28), ST (20), MD (20). Treatments are combined; conductivity above the CC line is excess.

Treatments CON, MW, MK, EW and EK were aged for 310 days under the conditions PR, ST and MD as in 2 mL volume of 2.5 mL flasks. The influence of ageing condition on conductivity at 25 and 5 °C is indicated in Figures 6 and 7 and the supplementary information. *S/V* was 3.9 cm^−1^. The results of conductivity measurements were compared with *σ*/*σ*_CC_ and were analyzed statistically using a linear mixed model (LMM) with regard to ageing conditions, treatment and temperature. The treatments were combined on the basis of the results in Figure 2.

**Figure 8:**
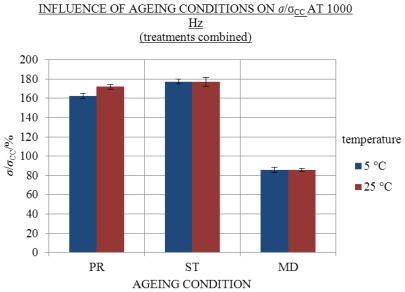
Influence of ageing conditions PR, ST and MD on average *σ*/*σ*_CC_ with SE intervals measured at 1000 Hz 25 (red) and 5 °C (blue) (treatments combined); number of replicate solutions, N (/): PR (28), ST (20), MD (20). Solutions aged for 310 days.

Under the conditions PR and ST approximately similar excess conductivity values were found at 25 and 5 °C, whereas under the MD condition no excess conductivity values were found (see Figures 6 and 7 and the supplementary information). The results show that the influence of ageing condition is significant, since *σ*/*σ*_CC_ under ST and PR conditions was essentially higher than under MD. In Table 1 and Figure 8 influence of ageing condition on *σ*/*σ*_CC_ are presented at 1000 Hz and 25 and 5 °C.

**Figure 9:**
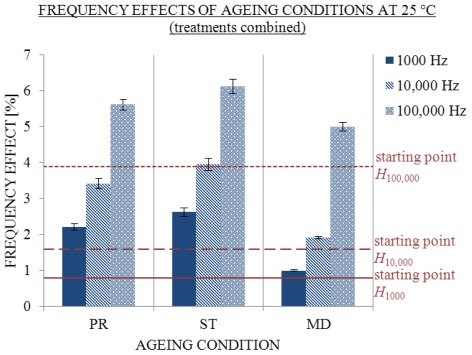
Influence of ageing condition on frequency effects *H; σ* measured at 1000 (filled), 10,000 (striped) and 100,000 Hz (dotted) 25 °C with SE intervals, when the treatments are combined. Conductivity was measured initially (starting point *H*) and on 310th day under PR, ST and MD conditions; number of replicate solutions, N (/): PR (28), ST (20), MD (20).

At both temperatures under the condition MD, *σ*/*σ*_CC_ was essentially lower than under PR and ST conditions (see Table 1 and Figure 8). The coefficients between measured and theoretical conductivity at 1000 Hz, *σ*/*σ*_UK1000_, of the same conditions measured at 25 and 5 °C had similar values (*p* = 0.734) (see the supplementary information). According to the similarity of the results, the conductivity values measured at 25 °C without a thermostat bath could be considered as valid. The frequency effects of the first and 310th day of ageing under different conditions are indicated in Figure 9.

At 25 °C the highest excess conductivity values related to condition ST, where the flasks were protected from daylight, followed by condition PR, where flasks were exposed to light; whereas under the condition MD, where the flasks were kept frozen, no excess values were found. The excess conductivities of conditions ST, PR and MD amounted to 43.3%, 41.8% and −15.6% of the measured conductivity *σ*, respectively. This means that the *σ*^E^ under conditions ST, PR and MD amounted to 20.7, 18.1 and −1.8 μS/cm, respectively (see Figure 6). The negative values for *σ*^E^ under the condition MD are ascribed to the time gap of ten days between the conductivity measurements and the ICP-MS analyses. At 25 and 5 °C the conditions PR and ST had approximately equal *σ*^E^ values (see Figures 6 and 7).

The results for conductivity values under condition MD are in accordance with our working hypothesis—the self-organizing abilities of water in the solid state are minimized due to diminished translational mobility. Namely, molecules of water in the solid state form a rigid structure. Besides, low temperatures under condition MD prevent release of alkaline ions, which could also be a reason for no excess conductivity values. While after 310 days under condition MD 0.01 mmol/L Na ± 1.8% (RSE) was leached, conditions ST and PR resulted in leaching of ten-times more, 0.16 mmol/L ± 5.5% and 0.14 mmol/L ± 3.1%, respectively.

Whereas in contrast to our assumption the excess conductivity values of solutions aged protected from daylight under condition ST were (when measuring temperatures and frequencies were combined) slightly, yet significantly (*p* = 0.003) higher than the *σ*^E^ of light-exposed flasks under condition PR (see Figure 8 and the supplementary information). Yet, for any conclusions on this topic more in-depth experiments are required.

Frequency effects exhibited a similar trend as *σ*/*σ*_CC_ (see Figure 9) and showed the relations between higher and lower frequencies of conductivity measurements. After 310 days of ageing, frequency effects were higher under all conditions than on the first day after treatment (starting point H). The greatest changes were found under the condition ST, followed by frequency effects under condition PR and minimal changes under condition MD, where no excess conductivity was found.

### 2.4. Influence of CO_2_ Absorption

**Figure 10:**
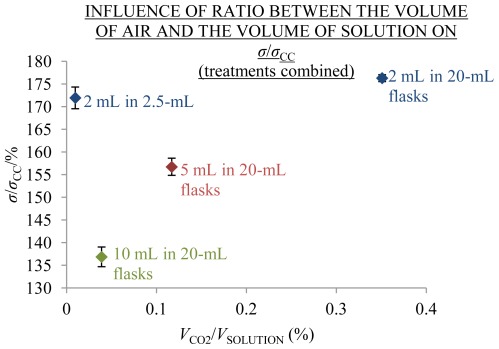
Average coefficients *σ*/*σ*_CC_ at 25 °C and 1000 Hz with SE intervals as a function of ratio of the volume of air above solution and the volume of solution (*V*_CO2_/*V*_SOLUTION_); treatments are combined. Solutions aged 310 or 370 days under the condition PR. Ordinate starts at 130%. N [/]: 0.01% of *V*_CO2_/*V*_SOLUTION_: 28; 0.04%: 10; 0.12%: 10; 0.35%: 8.

The samples measured had an approximately similar content of CO_2_ before bottling. Influence of CO_2_ absorption on conductivity of aged solutions was therefore approximately assumed by comparing the coefficients of measured and theoretical conductivity of solutions, aged 310 or 370 days under condition PR with different ratios of the volume of CO_2_ above solutions and the volume of solutions (*V*_CO2_/*V*_SOLUTION_). Above the 2 mL solutions in 2.5 and 20 mL flasks there were 0.5 and 18 mL of air with *V*_CO2_/*V*_SOLUTION_ 0.01 and 0.35, respectively. Whereas the *σ*/*σ*_CC_ of all aged 2 mL solutions were approximately equal and amounted to about 174% (see Table 2 and blue points in Figure 10). In addition, *σ*/*σ*_CC_ of 5 and 10 mL solutions after 370 days of ageing with *V*_CO2_/*V*_SOLUTION_ = 0.12 and 0.04, respectively, were significantly lower than *σ*/*σ*_CC_ of 2 mL solutions after 310 days of ageing with less *V*_CO2_/*V*_SOLUTION_ (0.01). From this we propose that CO_2_ absorption had no significant effect on the development of excess conductivity.

### 2.5. Influence of Ions on Time-Related Changes in Water Structure

**Figure 11:**
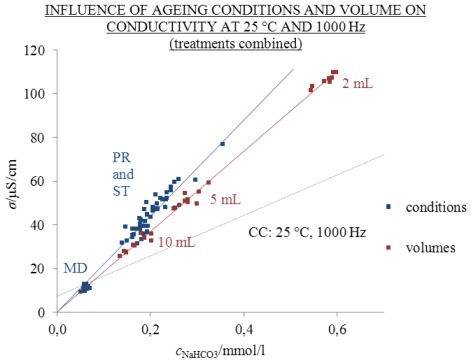
Conductivity at 25 °C and 1000 Hz as a function of *c*_NaHCO3_ of one day old (CC) and aged solutions. Ageing for 310 days in 2 mL of 2.5 mL flasks under conditions PR, ST and MD (blue) and ageing for 370 days in 2, 5 and 10 mL of 20 mL flasks (red). Points in the graph represent individual measurements. Conductivity above CC is excess.

Conductivity values measured at 1000 Hz were reproducible, therefore, all conductivity results at this frequency are combined in Figure 11 (the sum of Figures 3 and 6), where they are represented as a function of NaHCO_3_ concentration. For greater clarity, solutions aged in the three different volumes (2, 5 and 10 mL) are marked with the same colour (red), and all solutions aged under different conditions are marked with blue.

The linear trend line of the conductivity values of solutions aged in different volumes (Figure 3, red points in Figure 11) and the origin (0,0) gives the Equation (5) (with *R*^2^ = 0.9963):

(5) $$\sigma =184\times c_{\text{NaHCO}3}$$

On the other hand, the linear trend line through points for solutions aged under different conditions (Figure 6, blue points in Figure 11) and the origin gives the Equation (6) (with *R*^2^ = 0.9611):

(6) $$\sigma =220\times c_{\text{NaHCO}3}$$

Excess conductivity values in comparison to the conductivity of chemically analogous one-day-old solutions were found in all solutions aged for 310 and 370 days, except for the unfrozen samples (condition MD). This may indicate time related changes in the water structure. Both Equations (5) and (6) are linear (*R*^2^ are between 0.9611 and 0.9963) when going through the origin (0,0) despite ageing and, therefore, we may propose that both measured and excess conductivity values significantly depend on the concentration of dissolved NaHCO_3_. However, the question arises as to the function of dissolved ions in influencing the self-organizing abilities of water.

Vybíral and Voráček [19] noticed that water left to stand undisturbed for some time becomes autothixotropic, meaning that it spontaneously takes on gel-like properties. Because this phenomenon was not present in deionized water, they presume that ions in some way connected the water molecules into groups of “macroscopic” dimensions [18]. Turton and coworkers [24] reported optical Kerr effect and dielectric relaxation spectroscopic measurements of electrolyte solutions, which enabled them to separate the effects of rotational and translational motions of the water molecules. Their data show that electrolyte solutions behave like a supercooled liquid approaching the glass transition, in which rotational and translational molecular motions are decoupled.

Time related changes in water properties were also noticed by Lobyshev and coworkers [25]. They found that water emits weak luminescence in the range of the near ultraviolet and visible regions. The emission spectrum contains two wide bands that were proposed to depend on two factors: the ageing time of water in closed flasks and traces of luminescent and nonluminescent substances. They were lead to the conclusion that water and diluted aqueous solutions should be considered as polymorphous self-organizing systems.

## 3. Experimental Section

### 3.1. Materials

The solutes were Sigma-Aldrich or Merck products of the highest purity commercially available. The solutions of NaHCO_3_ were prepared using freshly prepared twice-distilled Milli-Q (Mq) water (Milli-Q water purification system, Millipore Corp.), with less than 2 μS/cm.

### 3.2. Procedures

#### 3.2.1. Mechanical Treatment

Mechanical treatment proceeded by iterating two steps: dilution (in our case centesimal, 1:100) and succussion. The process of succussion was carried out by vigorous mechanical agitation of the solution, in our case similar to the procedure of Elia and coworkers [6] with a vortex mixer at maximum power for 40 seconds. For vortexing, we used a Vibramix 10 from Tehtnica, Slovenia (50 W, 0.2 A) (3000 rpm, 50 Hz).

The starting point solution or the solvent for diluting was 0.05 or 0.1 mmol/L NaHCO_3_. For centesimal dilution (1C), a solute was dissolved in the solvent in a ratio of 1:100 and succussed. To get a 2C dilution, an aliquot of 1C was diluted in the solvent 1:100 and succussed. For example, for one centesimal dilution of KCl, 1 g of KCl was diluted with 99 g of the solvent, and then the solution was succussed. To prepare KCl 2C, the previous process was repeated using 1 g of KCl 1C and 99 g of solvent and the obtained solution was succussed. For 10C dilution, this process was repeated ten times, ending up with the original solute diluted by a factor of 10^−20^. Hence, the concentration of the original solute (KCl) in KCl 10C was equal to its concentration in the solvent. Therefore its physico-chemical properties should not differ from the solvent. Extremely dilute solutions of KCl and Mili-q water prepared in 0.05 or 0.1 mmol/L NaHCO_3_ were marked as MK and MW (KCl and Mq 10C), respectively (see Table 3).

#### 3.2.2. Electrical Treatment

**Figure 12:**
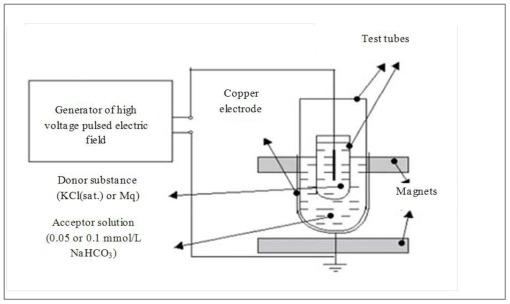
Scheme of electrical treatment of solutions.

For electrical treatment, a system consisting of a high voltage pulsed electric field source (23 kV, frequency of pulses 1.7 Hz) was constructed (see Figure 12).

The pulsed electric field modified by the electric field of a donor substance is supposed to change the physico-chemical properties of the acceptor solution [20]. The generator of high frequency pulses was connected to the 220 V network. The first pole of the generator was connected via a copper electrode and the second via a gold-plated wire that was placed into a 1.5 mL quartz test tube with a donor compound. Above and under the quartz test tube two permanent magnets were used to create a permanent magnetic field to increase the effectiveness of the treatment. The donor compounds were saturated aqueous solution of KCl or pure Milli-q water and the acceptor solutions (starting point solution) were 0.05 or 0.1 mmol/L NaHCO_3_. Seventy milliliters of starting point solution were poured into the acceptor tube. During the procedure the contents of the quartz test tube with the donor compound and the acceptor tube were not in physical contact. When as donor substances KCl (sat.) and Mq were used the treatments were marked as EK and EW, respectively (see Table 3).

### 3.3. Methods

#### 3.3.1. Conductivity Measurements

**Figure 13:**
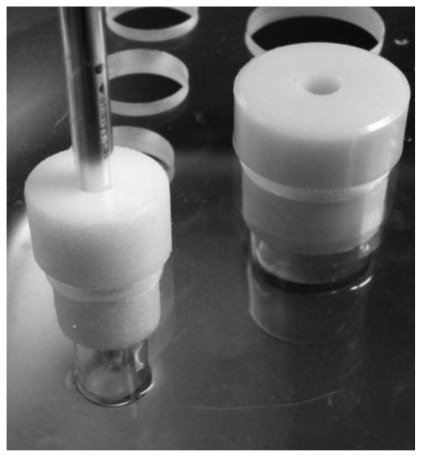
Teflon holders for inserting the measuring cell into 2.5 and 20 mL flasks.

Systematic measurements of electrical conductivity (μS/cm) were performed with a Stanford SR720 LCR impedance meter (Ronde & Schwartz). Four frequencies of alternating current were used: 120, 1000, 10,000 and 100,000 Hz. A grey platinized two-pole Microsamples CDC749 measuring cell (Radiometer Analytical) was used in the conductivity range 1–1000 μS/cm with a constant of 1.66 cm^−1^. Conductivity was measured in transparent 2.5-mL flasks (neoLab). For measurement at 5 °C, a VB13H thermostat (Kambič Laboratory Equipment d.o.o) with ± 0.1 °C accuracy and 10% NaCl (aq.) as thermostat bath were used. The impedance meter, thermostat and measuring cell were connected to the computer with help of Habe [26]. The measuring cell was inserted into the flasks with help of Teflon holders that enabled us to insert the measuring cell to the same depth and position of 2.5 or 20 mL flasks before each measurement and to prevent the contact of solutions with air (see Figure 13).

The cell constant was periodically checked using a standard KCl solution. Conductivity at 25 °C was measured in an air-conditioned laboratory room while the temperature was verified using a digital plug-in resistance thermometer with ± 0.01 °C accuracy. The conductivity values were temperature corrected using the temperature coefficient for conductivity 2%/°C. The differences in temperature did not exceed ± 1 °C. For the conductivity measurements at 5 °C, the flasks were thermostated using a 10% NaCl (aq.) thermostat bath where the temperature variation was ± 0.1 °C. The repeatability of the measuring system was tested by conductivity measurements of 10 replicate solutions of 0.5 mmol/L NaHCO_3_ at 25 °C whereby the measuring cell was removed and re-inserted into the flasks before each measurement. It proved to be ± 0.7% accurate.

#### 3.3.2. Analytical Determination of Impurities

The concentrations of Na, K, Mg, Ca and Ag in aged solutions were determined by an ICP-MS instrument (Agilent 7500ce). From the values obtained, the average of blank replicates (Mq, *N* = 6) was subtracted. The calibration curve for ICP-MS analyses was determined using the method of standard addition (*N* = 10). The detection limit was determined using the three-fold value of the blank samples (3s, *N* = 6) and amounted to 0.001 mg/L for Mg and 0.01 mg/L for Ca, Na and K.

### 3.4. Course of the Work

The starting point solution with a defined concentration of NaHCO_3_ (0.05 mmol/L or 0.1 mmol/L) was prepared. The conductivity of all treatments was measured directly after preparation and again after a certain amount of time. The solutions were aged in 2.5 mL flasks (neoLab) and 20 mL flasks (Wheaton) of transparent glass. During ageing the flasks were tightly closed. Since the glass of the flasks releases impurities, the solutions were analyzed for sodium, potassium, magnesium, silver and calcium by ICP-MS after the conductivity measurements were completed. The concentration of H_2_O_2_ in electrically and mechanically treated solutions was compared to the concentration in the Mq water using its chemical reaction with potassium permanganate.

**Figure 14:**
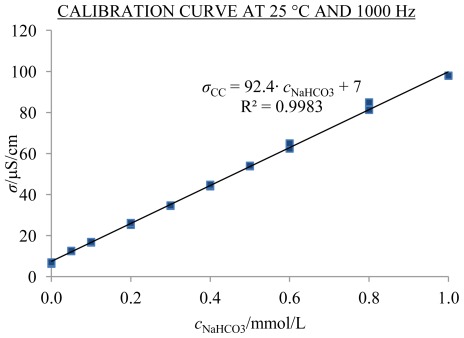
Calibration curve of NaHCO_3_ solutions (in duplicate) at 25 °C and 1000 Hz; points in the graph represent individual measurements.

Since according to the ICP-MS analyses the concentrations of K, Mg and Ca were approximately a tenth of that of Na, the former three ions were disregarded. The concentration of Ag was under the detection limit of the ICP-MS device. In aged solutions the concentration of H_2_O_2_ was very similar to the concentration in Mq. Silicon was analyzed by Elia *et al.* [8] by using UV-VIS spectroscopy and appeared in 100-fold smaller concentrations in comparison to Na. Therefore, the main chemical impurity in aged solutions due to the leaching of glass was sodium, which is released in the form of Na^+^ [27]. According to McGrail *et al.* [27], Na^+^ in the glass is exchanged for H^+^ or H_3_O^+^ ions whereas according to Elia *et al.* [10], OH^−^ reacts with dissolved CO_2_ into HCO_3_^−^. Therefore the chemical contribution of sodium to the conductivity or theoretically computed conductivity (*σ*_CC_) was, similarly to Elia *et al.* [10], computed using calibration curves in the following form: “*σ*_CC_ = k·*c*_NaHCO3_ + n” for four different frequencies (120, 1000, 10,000 and 100,000 Hz) at 25 °C and 5 °C (see Figure 14 and Tables 4 and 5). In this, the calibration curves were set up using freshly prepared one-day-old NaHCO_3_ solutions and the conductivity was measured in 2.5 mL flasks.

Similarly to Elia *et al.* [10], we found that experimental conductivity is a linear function of NaHCO_3_ concentration (see Table 5 and Figure 14). The calibration curve prepared by Elia *et al.* [10] at 25 °C and 2500 Hz is:

(7) $$\sigma \;[\mu \text{S}/\text{cm}]=93.7\cdot c_{\text{NaHCO}3}\;[\text{mmol}/\text{L}]+2$$

Equation (7) is similar to the calibration curve we obtained in 2.5 mL flasks at 1000 Hz and 10,000 Hz 25 °C (see Table 5). For preparation of solutions with different concentrations of NaHCO_3_, we used as solvent Mq water. Its conductivity value in the graphs of calibration curves was used at the concentration zero despite the fact that the conductivity of Mq water, measured in 2.5 mL flasks was approximately twice as high as when Mq water was measured in 20 mL flasks which reduces the calculated excess conductivity values by ~5 μS/cm.

Coefficients of the measured *σ* and theoretical conductivity values, *σ*/*σ*_CC_, presented as percentages were used for comparison of the following factors: ageing volume, condition and treatment and temperature of conductivity measurements. Namely, for excess conductivity values in some cases negative values were obtained. From *σ*/*σ*_CC_ we inferred the excess conductivity values (*σ*^E^) using the Equation (8):

(8) $$\sigma^{\text{E}}=\sigma -\sigma_{CC}$$

Frequency effects (*H*_f_) were calculated from an equation similar to that used by Shirai and Tamamushi [28], in that conductivity at 120 Hz was used as the reference conductivity: *H*_f_ = 100·(*σ*_f_ − *σ*^120^)/*σ*_120_. In this way the conductivity values at 1000, 10,000 and 100,000 Hz were compared to the reference conductivity at 120 Hz.

At 25 °C, when the conductivity was measured without a thermostat bath, the conductivity values slightly increased with increasing frequency, which is in agreement with Wachter and Barthel [29], while at 5 °C, when the conductivity was measured using a 10% NaCl (aq.) thermostat bath, due to side electric currents the conductivity values at 10,000 and 100,000 Hz in the concentration range of 0 to 0.1 mmol/L NaHCO_3_ were lower than at 120 Hz. The frequency effects at 1000 Hz were similar at both temperatures, therefore, at this frequency the conductivities of aged solutions were compared.

Before the experiments with aged solutions were performed, the repeatability of conductivity values after ageing for 310 days was tested by repeating the preparation (PR1 and PR2) twice and ageing of the five different treatments (see Table 3): CON, MW, MK, EW and EK. The two preparations, PR1 and PR2, were aged at two different time intervals for 310 days: PR1 (29 replicate solutions) was prepared at the end of May and the conductivity was measured at the beginning of April of the following year. PR2 (27 replicate solutions) was prepared at the beginning of March and the conductivity was measured in the middle of January of the following year.

Conductivity values at 1000 Hz of the two preparations were repeatable whereas the frequency effects of preparations PR1 and PR2 differed significantly (*p* < 0.001) and were not repeatable (see the supplementary information). Hence the conductivity values at 1000 Hz were compared in a common graph and the frequency effects were compared only within preparations—solutions aged in different volumes and under different conditions, separately. The relative standard error of frequency effects of 29 replicate solutions aged for 310 days did not exceed 4.5%.

Two series of experiments were conducted. In the first experiments conductivity was measured at 25 °C and in the second experiments at 5 °C as well. The *σ*/*σ*_CC_ values (*σ*^E^ = *σ* − *σ*_CC_) of the measurements at 5 and 25 °C at 1000 Hz did not differ significantly. From this we concluded that measurements without thermostat bath, at 25 °C, are reliable.

#### 3.4.1. Influence of Ageing Volume

In the first series of experiments, the influence of the ageing volume was tested using 2 mL, 5 mL and 10 mL solutions aged in 20 mL flasks for 370 days under the condition PR (exposed to daylight). The starting point solution was 0.05 mmol/L NaHCO_3_. The conductivity was measured at 25 °C in 2.5 mL flasks to which part of the contents of the 20 mL flasks was transferred.

#### 3.4.2. Influence of CO_2_ Absorption

Influence of CO_2_ absorption on conductivity was tested by comparing coefficients of measured and theoretical conductivity in solutions aged under the condition PR with different ratios between the volume of air and the volume of solution. Namely, 2 mL solutions in 2.5 and 20 mL flasks were in contact with 0.5 and 18 mL of air with approximately 8 and 290 nmol of CO_2_, respectively. The equilibrium concentration of CO_2_ in H_2_O (*c*_CO2_*) was according to the Equation (9):

(9) $${c_{\text{CO}2}}^{*}=\frac{y\;p\;c}{He}$$

where *He* is 1420 × 10^5^ Pa (Henry constant for CO_2_ in water at 20 °C), *y* is 0.00039 (molar share of CO_2_ in the air), *c* is 55.56 mol/L (average concentration of water), *p* is 1.013 × 10^5^ Pa and *c*_CO2_* is 15 μmol/L. Hence, at equilibrium there are 31 nmol of CO_2_ in 2 mL solutions. The flasks were tightly closed during the whole time of ageing. Therefore, if CO_2_ absorption were a significant factor influencing the conductivity, we propose that a significant difference between the coefficients of measured and theoretical conductivities of 2 mL solutions aged in 2.5 and 20 mL flasks should develop with time.

#### 3.4.3. Influence of Ageing Condition and Treatment

In the second series of experiments, the influence of ageing condition (see Table 6) and treatment were compared. The starting point solution, 0.05 mmol/L NaHCO_3_, corresponded to treatment MW, MK, EW and EK. All the treatments including the control (CON) were distributed in 2 mL portions into 2.5 mL flasks and aged under three different conditions. After 310 days the conductivity of the aged solutions was measured in the same flasks where the solutions were stored, at 25 and 5 °C.

## 4. Conclusions

- In accordance with Holandino *et al.*, but in contrast to Elia’s findings, previous treatment by mechanical shaking and repetitive dilution to extremely dilute solutions (as well as electric treatment with strong electrical impulses) performed in our laboratory had no significant influence on the conductivity of aged solutions.
- Significant excess conductivity values (*σ*^E^) compared to the conductivity of chemically analogous one-day-old solutions were found at 25 and 5 °C in all aged solutions except for those aged frozen at −20 °C.
- The excess conductivity values cannot be simply attributed to the absorption of CO_2_ due to the independency of *σ*/*σ*_CC_ from the ratio of the volume of air above solution and the volume of solution.
- The highest *σ*^E^ values at 25 °C were measured in 2 mL solutions aged under condition ST—protected from daylight for 310 days (20.3 μS/cm)—and 370 days under condition PR—exposed to daylight (46.0 μS/cm). The excess conductivity values cannot be attributed to low amounts of other ions disregarded in the calculations of the theoretical conductivity. Hence, we could probably ascribe the *σ*^E^ values to the ability of liquid water to spontaneously develop autothixotropic or gel-like properties, where ions and hydrophilic surfaces seem to play an important role. The autothixotropic properties enhance proton hopping mechanism in aqueous solutions and, therefore, increase the conductivity values.
- We assume that exclusion zones found previously by Pollack and coworkers [1–3] expanded with time from the surface to the bulk of the samples, which enhanced the proton hopping mechanism. Therefore, further experiments on this topic are planned.

## Supplementary Information



## Figures and Tables

**Table 1:** Average *σ*/*σ*_CC_ and relative standard errors (RSE) at 1000 Hz 25 and 5 °C of NaHCO_3_ solutions aged for 310 days in 2 mL volume of 2.5 mL flasks under conditions PR—exposed to daylight, ST—protected from daylight and MD—at −20 °C; treatments are combined.

Temperature (°C)	*σ/σ_CC_* (%) ± RSE (%)		

	PR	ST	MD
**25**	171.9% ± 1.4%	176.8% ± 2.6%	85.6% ± 1.8%
**5**	162.3% ± 1.5%	177.2% ± 1.5%	85.6% ± 3.2%

**Table 2:** Influence of dissolved CO_2_ (*V*_CO2_/*V*_SOLUTION_) on *σ*/*σ*_CC_ of aged solutions. Conductivity measured at 25 °C and 1000 Hz, treatments are combined.

Ageing	*V*_FLASK_	*V*_SOLUTION_	*V*_AIR_	*V*_CO2_/*V*_SOLUTION_	*S*/*V*	NaHCO_3_		*σ*/*σ*_CC1000_		

						AVG	SE	AVG	SE	N

d	mL	mL	mL	%	cm^−1^	mmol/L	mmol/L	%	%	/
310	2.5	2	0.5	0.01	3.9	0.19	0.01	171.9	2.4	28
370	20	2	18	0.35	4.0	0.58	0.01	176.3	0.8	8
370	20	5	15	0.12	2.6	0.28	0.01	156.7	1.9	10
370	20	10	10	0.04	2.1	0.17	0.01	136.8	2.2	10

**Table 3:** Used treatments.

Treatment	Donor	Abbreviation
no treatment—control	/	CON
mechanical treatment to 10C	Milli-q water	MW
	KCl	MK
electrical treatment	Milli-q water	EW
	KCl	EK

**Table 4:** Average conductivities of one-day-old NaHCO_3_ solutions (in duplicate) with deviations between the duplicates (Δ) measured at 25 and 5 °C and 1000 Hz.

*c*_NaHCO3_	*σ* (25 °C)	Δ (25 °C)	*σ* (5 °C)	Δ (5 °C)

mmol/L	μS/cm	μS/cm	μS/cm	μS/cm
0.05	12.5	0.1	7.5	0.0
0.10	16.8	0.2	10.5	0.1
0.20	25.7	0.5	16.3	0.2
0.30	34.6	0.1	22.1	0.0
0.40	44.4	0.4	28.0	0.4
0.50	53.9	0.2	33.6	0.2
0.60	63.7	1.3	39.5	0.0
0.80	83.1	1.8	50.7	0.3
1.00	98.0	0.0	/	/

**Table 5:** Equations of conductivity curves (CC) with deviations from linearity (*R*^2^); conductivity of one-day-old NaHCO_3_ solutions measured at 25 and 5 °C in 2.5 mL flasks. Conductivities, *σ*_CC_, are in μS/cm, concentrations, *c*_NaHCO3_, in mmol/L.

*T* (°C)	120 Hz	1000 Hz	10,000 Hz	100,000 Hz
25	*σ*_CC_*=* 90.1*c*_NaHCO3_ + 8	*σ*_CC_*=* 92.4*c*_NaHCO3_ + 7	*σ*_CC_*=* 93.4*c*_NaHCO3_ + 7	*σ*_CC_*=* 95.2*c*_NaHCO3_ + 8
	*R*^2^ = 0.9983	*R*^2^ = 0.9983	*R*^2^ = 0.9983	*R*^2^ = 0.9983
5	*σ*_CC_*=* 56.7*c*_NaHCO3_ + 5	*σ*_CC_*=* 58.1*c*_NaHCO3_ + 5	*σ*_CC_*=* 59.0*c*_NaHCO3_ + 4	*σ*_CC_*=* 56.0*c*_NaHCO3_ + 2
	*R*^2^ = 0.9996	*R*^2^ = 0.9997	*R*^2^ = 0.9996	*R*^2^ = 0.9973

**Table 6:** Ageing conditions.

Condition	Influences
**PR**	exposed to daylight
**ST**	protected from daylight
**MD**	at low temperatures: −20 °C
