# Supplementary material for: Possible Time-Dependent Effect of Ions and Hydrophilic Surfaces on the Electrical Conductivity of Aqueous Solutions
Source: Int J Mol Sci. 2012 Mar 27;13(4):4048–68. doi: 10.3390/ijms13044048 (PMC3344201; doi:10.3390/ijms13044048)
Supplement: Supplementary file 1 [file ijms-13-04048-s001.pdf]

Supplementary Information

Possible Time-Dependent Effect of Ions and Hydrophilic Surfaces on the Electrical Conductivity of Aqueous Solution

Nada Verdel [1,]*, Igor Jerman [1], Rok Krašovec [1], Peter Bukovec [2] and Marija Zupančič [2]

[1] Institute Bion d. o. o., Stegne 21, 1000 Ljubljana, Slovenia; E-Mails: igor.jerman@bion.si (I.J.); rok.krasovec@bion.si (R.K.)

[2] Department of Chemistry, University of Ljubljana, Aškerčeva 5, 1000 Ljubljana, Slovenia; E-Mails: peter.bukovec@fkkt.uni-lj.si (P.B.); marija.zupancic@fkkt.uni-lj.si (M.Z.)

***** Author to whom correspondence should be addressed; E-Mail: nada.verdel@bion.si; Tel.: +386-41-959-546; Fax: +386-1-513-11-47.

Received: 21 January 2012; in revised form: 28 February 2012 / Accepted: 13 March 2012 / Published: 27 March 2012

Abstract: The purpose of this work was to determine the influence of mechanical and electrical treatment on the electrical conductivity of aqueous solutions. Solutions were treated mechanically by iteration of two steps: 1:100 dilution and vigorous shaking. These two processes were repeated until extremely dilute solutions were obtained. For electrical treatment the solutions were exposed to strong electrical impulses. Effects of mechanical (as well as electrical) treatment could not be demonstrated using electrical conductivity measurements. However, significantly higher conductivity than those of the freshly prepared chemically analogous solutions was found in all aged solutions except for those samples stored frozen. The results surprisingly resemble a previously observed weak gel-like behaviour in water stored in closed flasks. We suggest that ions and contact with hydrophilic glass surface could be the determinative conditions for the occurrence of this phenomenon.

Keywords: autothixotropy; conductivity; exclusion zone; extremely dilute solutions; water

---

1. Experimental

1.1. Introduction to the Conductivity Measuring System

Due to the use of platinized electrodes and dilute solutions, some measurements were inconsistent, so the average of ten replicate measurements of the sample was calculated and only if the relative standard error of the ten measurements were less than 1%, was the average considered as a valid result (see Table S.1).

Table S.1. Example of ten conductivity measurements of 104 μmol/L $NaHCO_3$ at 120, 1000, 10,000 and 100,000 Hz and 5 °C with descriptive statistics—averages (AVG), standard deviations (SD), standard errors (SE) and relative standard errors (RSE).

**Descriptive Statistics**		σ			
		**120 Hz**	**1000 Hz**	**10,000 Hz**	**100,000 Hz**
1.	σ/ μS/cm	10.7	10.8	10.6	8.0
2.	σ/ μS/cm	10.7	10.8	10.5	8.0
3.	σ/ μS/cm	10.6	10.7	10.5	7.9
4.	σ/ μS/cm	10.6	10.7	10.4	7.9
5.	σ/ μS/cm	10.5	10.6	10.4	7.8
6.	σ/ μS/cm	10.5	10.6	10.3	7.8
7.	σ/ μS/cm	10.4	10.5	10.3	7.8
8.	σ/ μS/cm	10.4	10.5	10.3	7.8
9.	σ/ μS/cm	10.4	10.5	10.2	7.7
10.	σ/ μS/cm	10.4	10.4	10.2	7.7
AVG/ μS/cm		10.5	10.6	10.4	7.8
SD/ μS/cm		0.1	0.1	0.1	0.1
SE/ μS/cm		0.04	0.04	0.04	0.03
RSE/%		0.37	0.41	0.41	0.42

Before measuring the conductivity of samples, the calibration curves, σ_{CC} = f(c_{NaHCO_3}), were prepared. From the calibration curves the theoretical conductivity was calculated. Because in some samples the excess conductivity values, σ^E, were negative, for the statistical outcome coefficients between the measured and theoretical conductivity as a percentage were also used (σ/σ_{CC}). From σ/σ_{CC} the excess conductivity values were inferred as follows:

$$\sigma/\sigma_{CC} = \sigma/(\sigma - \sigma^E) \quad \text{(S.1)}$$

If $\sigma/\sigma_{CC} = 1.80 = \sigma/(\sigma - \sigma^E)$, then $\sigma^E = \sigma \times 0.80/1.80 = \sigma \times 0.44$. This means that 44% of measured conductivity is excess.

The measuring cell was regularly calibrated by means of a 0.0002 M standard KCl solution, which was prepared as follows: KCl was dried at 180 °C for two and half hours and cooled overnight in a desiccator. 0.7545 g of dried KCl was dissolved in 1 litre Mq water with specific conductivity of less than 2 μS/cm and diluted 1:50. The conductivity values of KCl solution at 1000 Hz and 20 °C were compared to the ICUMSA conductivity value [1] (26.6 ± 0.3 μS/cm).

The repeatability of the measuring system was tested by conductivity measurements of 10 replicates of 0.5 mmol/L $NaHCO_3$ at 25 °C. Before each measurement the measuring cell was removed and re-inserted into the flask. Each measurement was repeated ten times and SD, SE and RSE were calculated.

To evaluate the influence of the dielectric constant of the thermostat bath on the conductivity measurements at higher frequencies, the conductivity values at 10,000 and 100,000 Hz of three systems with thermostat baths of different dielectric constants were compared. As thermostat baths, distilled water with ε~80, edible oil with ε~2.5 and air with ε~1 were used (see Figure S.1). Frequency effects on the conductivity measurements were compared with triplicate solutions of 0.5 mmol/L $NaHCO_3$, measured in 20-mL flasks.

Figure S.1. Systems with thermostat baths of different dielectric constants: (**a**) water bath with ε~80, (**b**) oil bath with ε~2.5 and (**c**) without thermostat bath with ε~1.

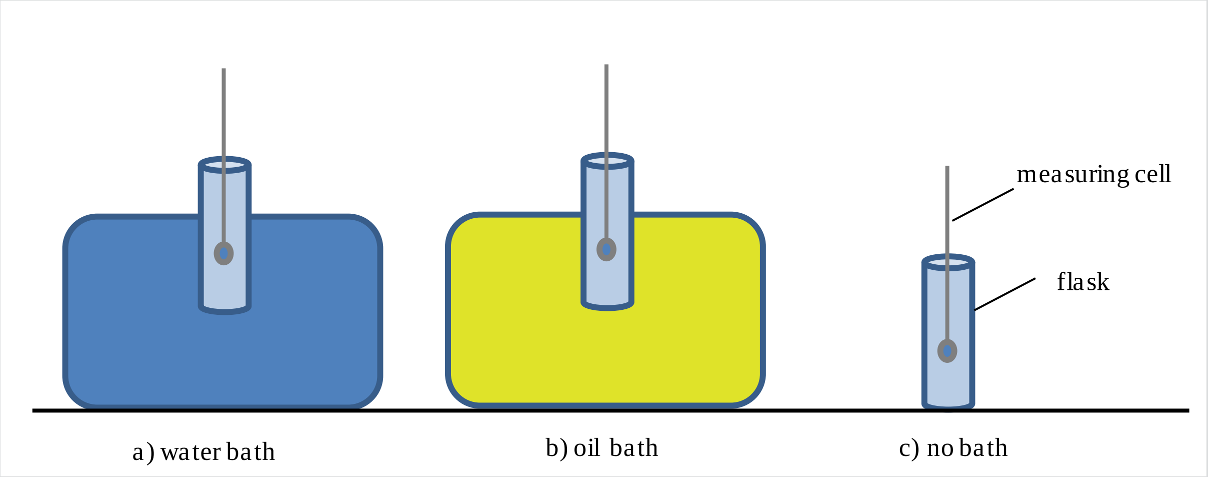

The $NaHCO_3$ conductivity calibration curve was prepared by measuring the conductivity of different concentrations of $NaHCO_3$ in 2.5-mL flasks. The solutions were prepared by diluting 0.05 mol/L $NaHCO_3$ with Mq water (see Table S.2).

Table S.2. Preparation of solutions for conductivity calibration curve from 0.05 mol/L $NaHCO_3$ and Mq water.

$V_{0.05\ mol/L NaHCO3}$ mL	V_{Mq} mL	c_{NaHCO3} mmol/L
0.48	19.52	1.2
0.40	19.60	1.0
0.32	19.68	0.8
0.24	19.76	0.6
0.20	19.80	0.5
0.16	19.84	0.4
0.12	19.88	0.3
0.08	19.92	0.2
0.04	19.90	0.1
0.02	19.98	0.05

From the graphs of the dependence of conductivity on $NaHCO_3$ concentration, 8 calibration curves were calculated, four at 25 ℃ and 4 at 5 ℃.

1.2. Testing the Working Hypothesis

The working hypothesis was tested by conductivity measurements in 2.5-mL flasks at four frequencies: 120, 1000, 10,000 and 100,000 Hz. Firstly, the repeatability of solution preparation and treatment was determined. Secondly, the influence of the ageing volume was determined. Thirdly, the dependence of conductivity on ageing conditions, treatment (*i.e.*, mechanical or electric) and temperature was examined.

1.2.1. Repeatability of Ageing

The repeatability of the conductivity of aged solutions was checked by two repetitions of the preparation and ageing treatments CON, MW, MK, EW and EK (see Table S.3). The two preparations PR1 and PR2 were aged at different time periods for 310 days: PR1 was prepared and treated on the 20th of May 2009, PR2 on the 2nd of March 2009 and the conductivity of both preparations was measured after 310 days. The conductivity of the prepared solutions at 1000 Hz was compared by univariate analysis (UA) and the linear mixed model (LMM) according to preparation and treatment; see Table S.4. Both statistical tests gave similar results.

Table S.3. Repeatability of ageing of preparations PR1 and PR2—descriptions of treatments CON, MW, MK, EW and EK, with numbers of replicates (N).

Preparation	N	Treatment
PR1	5	CON—untreated 0.05 mmol/L $NaHCO_3$
	5	MW—10C, source of »information« Mq, succussion by vibration mixer—same concentration of Mq as in the starting point solution
	7	MK—10C, source of »information« KCl (s), succussion by vibration mixer—same concentration of KCl as in the starting point solution
	5	EW—source of »information« Mq, duration of electric treatment 25 minutes—same concentration of Mq as in the starting point solution
	7	EK—source of »information« KCl (sat.), duration of electric treatment 25 minutes—same concentration of KCl as in the starting point solution
PR2	7	CON—untreated 0.1 mmol/L $NaHCO_3$
	5	MW—10C, source of »information« Mq, succussion by vibration mixer—same concentration of Mq as in the starting point solution
	5	MK—10C, source of »information« KCl (s), succussion by vibration mixer—same concentration of KCl as in the starting point solution
	5	EW—source of »information« Mq, duration of electric treatment 25 minutes—same concentration of Mq as in the starting point solution
	5	EK—source of »information« KCl (sat.), duration of electric treatment 25 minutes—same concentration of KCl as in the starting point solution

Table S.4. Repeatability of ageing; statistical data.

Statistical Factors	Conductivity at 1000 Hz
studied factors	1. preparation (PR1, PR2)<br>2. treatment (CON, MW, MK, EW, EK)
interactions between factors	preparation and treatment
disturbing factors	different initial concentrations of $NaHCO_3$ in the starting point solutions
experimental units	2.5-mL flasks
number of replicates	PR1: 29, PR2: 27
outcome of the experiment	conductivity σ_{1000}
normal distribution	no
variances	homogeneous
statistical analysis with LMM	subject: flask; dependent variable: σ_{1000}; factor: preparation, treatment; full factorial analysis;
statistical analysis with UA	dependent variable: σ_{1000}; fixed factor: preparation, treatment;

1.2.2. Influence of Ageing Volume

The influence of ageing volume was tested using 2, 5 and 10 mL aged for 370 days in 20-mL flasks under the condition PR (exposed to daylight). The starting point solution was 0.05 mmol / L $NaHCO_3$. The conductivity was measured at 25 °C in 2.5-mL flasks to which part of the content of 20-mL flasks was transferred. The $NaHCO_3$ concentration of solutions aged in different volumes differed significantly. Therefore the influence of ageing volume was determined by comparing σ/σ_{CC} at 1000 Hz and frequency effects at 1000, 10,000 and 100,000 Hz.

1.2.3. Influence of Ageing Condition, Treatment and Temperature

Effects of condition and treatment on ageing was checked by ageing of five treatments, CON, MW, MK, EW and EK, under three conditions, PR, ST and MD (see Table 4 in the manuscript) for 310 days. The starting point solution, 0.05 mmol/L $NaHCO_3$, was treated in the same manner as the preparation PR1 (see Table S.3). Treatments and controls were divided into 2.5-mL flasks and aged under the three different conditions. After 310 days the conductivity of solutions was measured by immersing the measuring cell into the flasks where they were stored. The conductivity was measured at 25 and 5 °C. Treatments were marked as CON, MW, MK, EW and EK and compared with σ/σ_{CC} at 1000 Hz and frequency effects at 1000, 10,000 and 100,000 Hz.

2 Results

2.1. Introduction to the Conductivity Measuring System

2.1.1. Accuracy of Conductivity Measurements

The repeatability of conductivity measurements was tested by measuring ten replicate solutions of 0.5 mmol/L $NaHCO_3$. Before each measurement the measuring cell was removed and re-inserted into the flask (see Table S.5). The conductivity of each solution was measured ten times and the average value was considered as the result, if the RSE was less than 1%.

Table S.5. Repeatability of the conductivity of 10 replicate solutions of 0.5 mmol/L $NaHCO_3$ measured at 25 °C and four frequencies in 20-mL flasks. Descriptive statistics of conductivity with AVG, SD, SE, N and RSE is added.

***f*/Hz**	**120**	**1000**	**10,000**	**100,000**
AVG σ/ µS/cm	50.5	50.6	51.2	51.9
SD/ µS/cm	0.97	0.97	1.03	1.20
N/	10	10	10	10
SE/ µS/cm	0.31	0.31	0.33	0.38
RSE/%	0.6	0.6	0.6	0.7

The highest relative standard error (RSE) of the conductivity measurements of ten replicates of 0.5 mmol/L $NaHCO_3$, at 25 °C and at four frequencies by removal and re-insertion of the measuring cell into the flasks was 0.7%.

2.1.2. Influence of ε of the Thermostatic Bath on Conductivity Values at 10 and 100 kHz

We noticed that the conductivity values at 10,000 and 100,000 Hz strongly depend on the dielectric constant of the thermostat bath into which the measuring vials are placed during the measurements. We therefore measured the conductivity of 0.5 mmol/L $NaHCO_3$ in 20-mL flasks in three measurement systems that differed in the dielectric constant of the thermostat bath (see Figure S.2). For each system triplicates solutions were measured (see Tables S.6 and S.7).

Figure S.2. Frequency effects with standard error intervals at 1000 (filled), 10,000 (striped) and 100,000 Hz (dotted) in 0.5 mmol/L $NaHCO_3$ measured in systems with baths of different dielectric constants; water (ε = 80), oil bath (ε = 2.5) or without bath (ε = 1), at 25 °C.

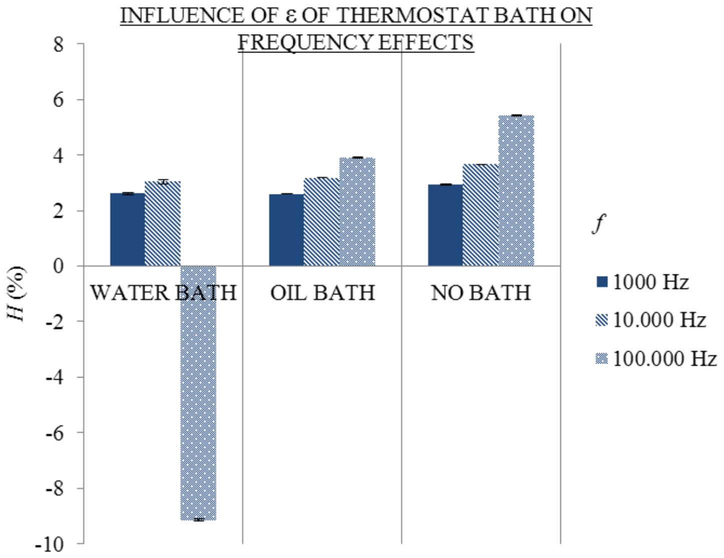

Table S.6. Conductivity values of three replicates of 0.5 mmol/L $NaHCO_3$ measured at 25 °C in water, an oil bath and without a thermostat bath.

System	*f*/Hz	120	1000	10,000	100,000
water bath	AVG σ/ µS/cm	68.9	70.7	71.0	62.6
	SD/ µS/cm	0.68	0.66	0.64	0.66
	SE/ µS/cm	0.39	0.38	0.37	0.38
	RSE/%	0.6	0.5	0.5	0.6
oil bath	AVG σ/ µS/cm	69.1	70.9	71.3	71.8
	SD/ µS/cm	0.21	0.22	0.23	0.23
	SE/ µS/cm	0.12	0.13	0.13	0.13
	RSE/%	0.2	0.2	0.2	0.2
no bath	AVG σ/ µS/cm	68.2	70.2	70.7	71.9
	SD/ µS/cm	0.93	0.97	0.98	0.99
	SE/ µS/cm	0.54	0.56	0.57	0.57
	RSE/%	0.8	0.8	0.8	0.8

Table S.7. Frequency effects (H) at 1000, 10,000 and 100,000 Hz of triplicate solutions of 0.5 mmol/L $NaHCO_3$ in systems with a water bath or oil bath or without a thermostat bath, at 25 °C.

System	*ε*/	H_{1000}/%	$H_{10,000}$/%	$H_{100.000}$/%
water bath	80	2.6	3.0	−9.1
oil bath	2.5	2.6	3.2	3.9
no bath	1	2.9	3.7	5.4

Conductivity values at 100,000 Hz significantly differed between the three systems (see Figure S.2). Without the thermostat bath, when the flasks were only in contact with air, the conductivity values increased with rising frequency. At 1000 Hz the frequency effect was 2.9%, at 10,000 Hz 3.7% and at 100,000 Hz 5.4% (see Table S.7). In the oil bath (the flask was immersed in a beaker filled with oil and placed in a thermostat bath filled with distilled water) with ε~2.5, the increase of conductivity with increasing frequency was weaker. The frequency effect at 1000 Hz was 2.6%, at 10,000 Hz 3.2% and at 100,000 Hz 3.9%. While in the water bath (the flask was immersed into distilled water in the thermostat bath) with ε = 80, the conductivity at 1000 Hz grew by 2.6%, at 10,000 Hz by 3.0% and while at 100,000 Hz the conductivity values were smaller than at 120 Hz by ~9.1%.

In order to minimize external influences on the measurement of conductivity at 10,000 and 100,000 Hz NaCl was added (w_{NaCl} = 10%) to distilled water in the thermostat bath.

2.1.3. Content of Sodium, Potassium, Magnesium and Calcium in 310 Days Aged Solutions

We found that aged samples had significantly more sodium than potassium, calcium and magnesium and therefore those latter three elements were excluded from calculations of the theoretical conductivity values (see Table S.8).

Table S.8. ICP-MS analysis of sodium, potassium, calcium and magnesium in solutions after 310 days under different ageing conditions (PR, ST and MD)—for details of ageing conditions see manuscript, Table 4.

Ion	Descriptive Statistics	PR	ST	MD
Na	**AVG *c*/mmol/L**	**1.94×10^{-1}**	**2.14×10^{-1}**	**6.15×10^{-2}**
	SD/mmol/L	3.15×10^{-2}	5.23×10^{-2}	4.89×10^{-3}
	N	28	20	20
	SE/mmol/L	5.96×10^{-3}	1.17×10^{-2}	1.09×10^{-3}
	RSE/%	3.1	5.5	1.8
K	**AVG *c*/mmol/L**	**3.13×10^{-2}**	**3.80×10^{-2}**	**7.89×10^{-3}**
	SD/mmol/L	2.23×10^{-3}	5.51×10^{-3}	9.05×10^{-4}
	N	29	21	21
	SE/mmol/L	3.53×10^{-3}	5.17×10^{-3}	6.25×10^{-4}
	RSE/%	11.32	13.66	7.74
Ca	**AVG *c*/mmol/L**	**2.51×10^{-2}**	**2.53×10^{-2}**	**1.36×10^{-2}**
	SD/mmol/L	2.74×10^{-3}	3.27×10^{-3}	3.34×10^{-3}
	N	29	21	21
	SE/mmol/L	3.37×10^{-3}	3.89×10^{-3}	1.67×10^{-3}

Table S.8. *Cont.*

Ion	Descriptive Statistics	PR	ST	MD
Ca	RSE/%	13.58	15.34	14.42
	**AVG *c*/mmol/L**	**8.70×10^{-3}**	**1.46×10^{-2}**	**2.60×10^{-3}**
	SD/mmol/L	1.09×10^{-3}	3.64×10^{-3}	6.47×10^{-5}
Mg	N	29	21	21
	SE/mmol/L	1.14×10^{-3}	2.34×10^{-3}	1.83×10^{-3}
	RSE/%	13.08	15.96	7.08

2.1.4. Determination of Hydrogen Peroxide

Mechanically and electrically treated aged samples had the same concentration of hydrogen peroxide as the Mq water used as solvent (see Table S.9). Therefore H_2O_2 was disregarded in calculation of the theoretical conductivity.

Table S.9. Concentrations of hydrogen peroxide in 310 days aged treatments MW, EW and EK and in blind replicates (Mq); for details on treatments see manuscript Table 1.

Descriptive Statistics	Mq	MW	EW	EK
**AVG c_{H2O2}/µmol/L**	**1.05**	**1.05**	**1.05**	**1.05**
ageing /d	1	524	524	524
SD/µmol/L	0.01	0.00	0.00	0.00
N	3	25	25	25

2.1.5. Calibration Curves for the Conductivity of $NaHCO_3$

The relation between concentration and conductivity values was determined by measuring the conductivity of solutions with precisely known composition. A calibration curve (CC) was drawn as the conductivity as a function of concentration. In Tables A10 and A11 the conductivity values of 0 to 1 mmol/L $NaHCO_3$ solutions (in duplicate), measured at 25 and 5 °C and the differences in conductivity values between the duplicate solutions are listed, respectively. In Table S.12 calibration equations used for calculating the theoretical conductivity values with deviations from linearity (R^2) are given. At 25 °C solutions were measured in a thermostated room without a thermostat bath, so the conductivity values were temperature adjusted to 25 °C using a plug-in thermometer with ± 0.01 °C accuracy and a conductivity coefficient of 2%/°C. Deviations from 25 °C were maximally ± 1 °C. At 5 °C the conductivity of the solutions was measured by immersing the flasks in a thermostat bath with 10% NaCl. Deviations from 5 °C were ± 0.1 °C.

Table S.10. Conductivities of fresh $NaHCO_3$ solutions at 25 and 5 °C.

c_{NaHCO3}	T	N	σ_{120}	σ_{1000}	$\sigma_{10,000}$	$\sigma_{100,000}$
**mmol/L**	**°C**	/	**µS/cm**	**µS/cm**	**µS/cm**	**µS/cm**
0.00	25	2	6.6	6.6	6.7	7.1
0.05	25	2	12.4	12.5	12.6	12.9
0.10	25	2	16.6	16.8	16.9	17.3

Table S.10. *Cont.*

c_{NaHCO_3}	*T*	N	σ_{120}	$\sigma_{1,000}$	$\sigma_{10,000}$	$\sigma_{100,000}$
**mmol/L**	**°C**	/	**µS/cm**	**µS/cm**	**µS/cm**	**µS/cm**
0.20	25	2	25.4	25.7	25.9	26.5
0.30	25	2	34.1	34.6	34.9	35.6
0.40	25	2	43.7	44.4	44.9	45.8
0.50	25	2	52.9	53.9	54.6	55.6
0.60	25	2	62.3	63.7	64.4	65.7
0.80	25	2	81.3	83.1	84.0	85.7
1.00	25	2	95.7	98.0	98.9	101.0
0.00	5	2	4.1	4.1	3.6	3.0
0.05	5	2	7.4	7.5	7.2	5.4
0.10	5	2	10.4	10.5	10.3	7.7
0.20	5	2	16.1	16.3	16.2	12.7
0.30	5	2	21.8	22.1	22.1	17.8
0.40	5	2	27.6	28.0	28.1	23.4
0.50	5	2	33.0	33.6	33.8	29.1
0.60	5	2	38.8	39.5	39.8	35.3
0.80	5	2	49.7	50.7	51.2	47.1

Table S.11. Differences in conductivity values between duplicates (△).

c_{NaHCO_3}	*T*	N	$\triangle_{120}$	$\triangle_{1,000}$	$\triangle_{10,000}$	$\triangle_{100.000}$
**mmol/L**	**°C**	/	**µS/cm**	**µS/cm**	**µS/cm**	**µS/cm**
0.00	25	2	0.3	0.5	0.4	0.3
0.05	25	2	0.1	0.1	0.1	0.2
0.10	25	2	0.1	0.2	0.2	0.2
0.20	25	2	0.5	0.5	0.5	0.5
0.30	25	2	0.1	0.1	0.1	0.1
0.40	25	2	0.4	0.4	0.4	0.4
0.50	25	2	0.2	0.2	0.2	0.2
0.60	25	2	1.2	1.3	1.3	1.3
0.80	25	2	1.8	1.8	1.9	1.9
1.00	25	2	0.0	0.0	0.1	0.1
0.00	5	2	0.1	0.1	0.1	0.1
c_{NaHCO_3}	*T*	N	$\triangle_{120}$	$\triangle_{1,000}$	$\triangle_{10,000}$	$\triangle_{100.000}$
**mmol/L**	**°C**	/	**µS/cm**	**µS/cm**	**µS/cm**	**µS/cm**
0.05	5	2	0.0	0.0	0.0	0.0
0.10	5	2	0.1	0.1	0.1	0.2
0.20	5	2	0.2	0.2	0.2	0.4
0.30	5	2	0.0	0.0	0.1	0.0
0.40	5	2	0.5	0.4	0.4	0.4
0.50	5	2	0.2	0.2	0.2	0.1
0.60	5	2	0.0	0.0	0.0	0.0
0.80	5	2	0.3	0.3	0.3	0.2
1.00	5	1	0.0	0.0	0.0	0.0

Table S.12. Calibration equations with deviations from linearity (R^2); conductivity of one day old $NaHCO_3$ solutions measured at 25 and 5 °C. Conductivities, σ_{CC}, are in µS/cm, concentrations, c_{NaHCO3}, in mmol/L.

T (°C)	Description	120 Hz	1000 Hz	10,000 Hz	100,000 Hz
25	equation	$\sigma_{CC} = 90.1\ c_{NaHCO3} + 8$	$\sigma_{CC} = 92.4\ c_{NaHCO3} + 7$	$\sigma_{CC} = 93.4\ c_{NaHCO3} + 7$	$\sigma_{CC} = 95.2\ c_{NaHCO3} + 8$
	R^2	0.9983	0.9983	0.9983	0.9983
5	equation	$\sigma_{CC} = 56.7\ c_{NaHCO3} + 5$	$\sigma_{CC} = 58.1\ c_{NaHCO3} + 5$	$\sigma_{CC} = 59.0\ c_{NaHCO3} + 4$	$\sigma_{CC} = 56.0\ c_{NaHCO3} + 2$
	R^2	0.9996	0.9997	0.9996	0.9973

Mq water was used as solvent so its conductivity value was used as the concentration zero. Despite the fact that the conductivity of Mq water, measured in 2.5-mL flasks was approximately twice as high as when Mq water was measured in 20-mL flasks, we took the conductivity values when measured in 2.5-mL flasks into account in calculations of the calibration curve, through which the excess conductivity values were reduced by ~5 µS/cm. Elia *et al.* [2] published conductivity values of $NaHCO_3$ solutions at 25 °C and 2.5 kHz from which the following calibration equation was calculated:

$$\sigma\ [\mu S/cm] = 93.7 \times c_{NaHCO3}\ [mmol/L] + 2 \quad (S.2)$$

which is similar to the calibration equation obtained from our measurement system in 2.5-mL flasks at 1 kHz and 25 °C (see Table S.12):

$$\sigma\ [\mu S/cm] = 92.4 \times c_{NaHCO3}\ [mmol/L] + 7 \quad (S.3)$$

or at 10 kHz and 25 °C (see Table S.12):

$$\sigma\ [\mu S/cm] = 93.4 \times c_{NaHCO3}\ [mmol/L] + 7 \quad (S.4)$$

Here we illustrate only two of the eight CC owing to the similarity of the CC graphs at all the used frequencies and temperatures (see Figures S.3 and S.4).

Figure S.3. CC of $NaHCO_3$ solutions (in duplicate), conductivity measured at 25 °C and 1000 Hz. Points in the graph represent individual measurements.

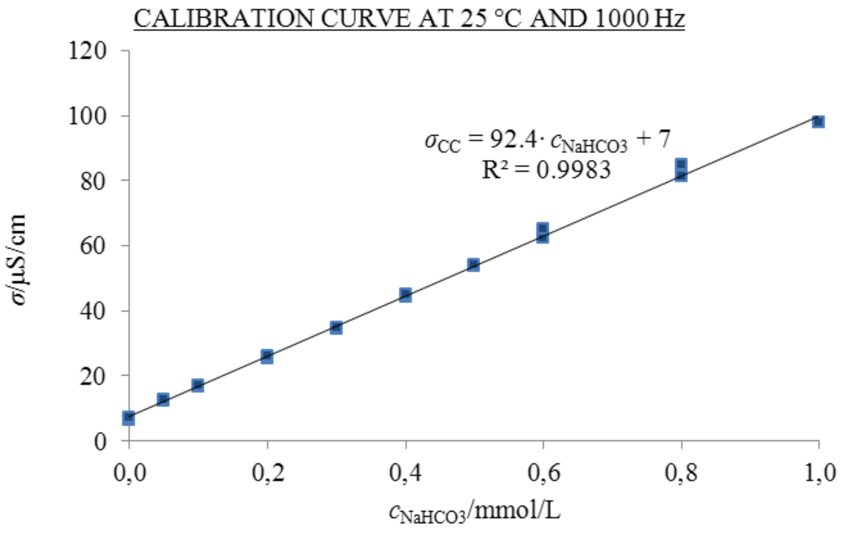

Figure S.4. CC of $NaHCO_3$ solutions (in duplicate), conductivity measured at 5 ℃ and 1000 Hz. Points in the graph represent individual measurements.

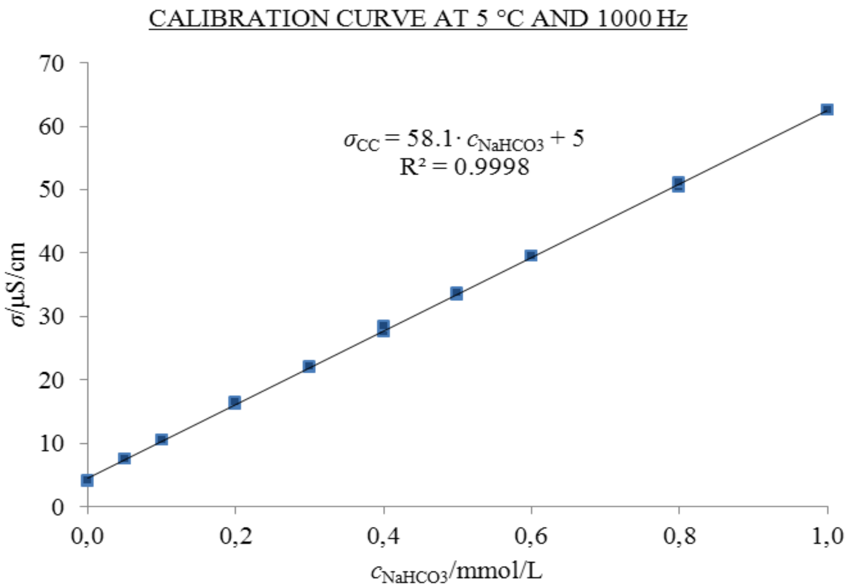

Conductivity values at 100,000 Hz and 25 ℃ increased with increasing frequency, while the conductivity values at 5 ℃ increased with increasing frequency up to 10,000 Hz, while at 100,000 Hz they fell below the value at 120 Hz, supposedly due to adverse side electric currents along the cell wall (see Table S.10). The conductivity values at 25 and 5 ℃ (measured at 100,000 Hz) therefore cannot be compared directly. The composition of the thermostat bath used for the conductivity measurements of fresh (one day old) and aged solutions of $NaHCO_3$ was kept as equal as possible to counterbalance the influence of the surrounding currents.

2.1.6 Frequency Effects

Using the results of the conductivity measurements of the $NaHCO_3$ solutions prepared for CC, the frequency effects at different $NaHCO_3$ concentrations were calculated using the following equation (see Table S.13 and Figures S.5 and S.6):

$$H_f = 100\,(\sigma_f - \sigma_{120})/\sigma_{120} \qquad \text{(A5)}$$

Figure S.5. Frequency effects with intervals of differences between duplicates at 1000 (filled), 10,000 (striped) and 100,000 Hz (dotted) of one day old $NaHCO_3$ solutions at 25 ℃.

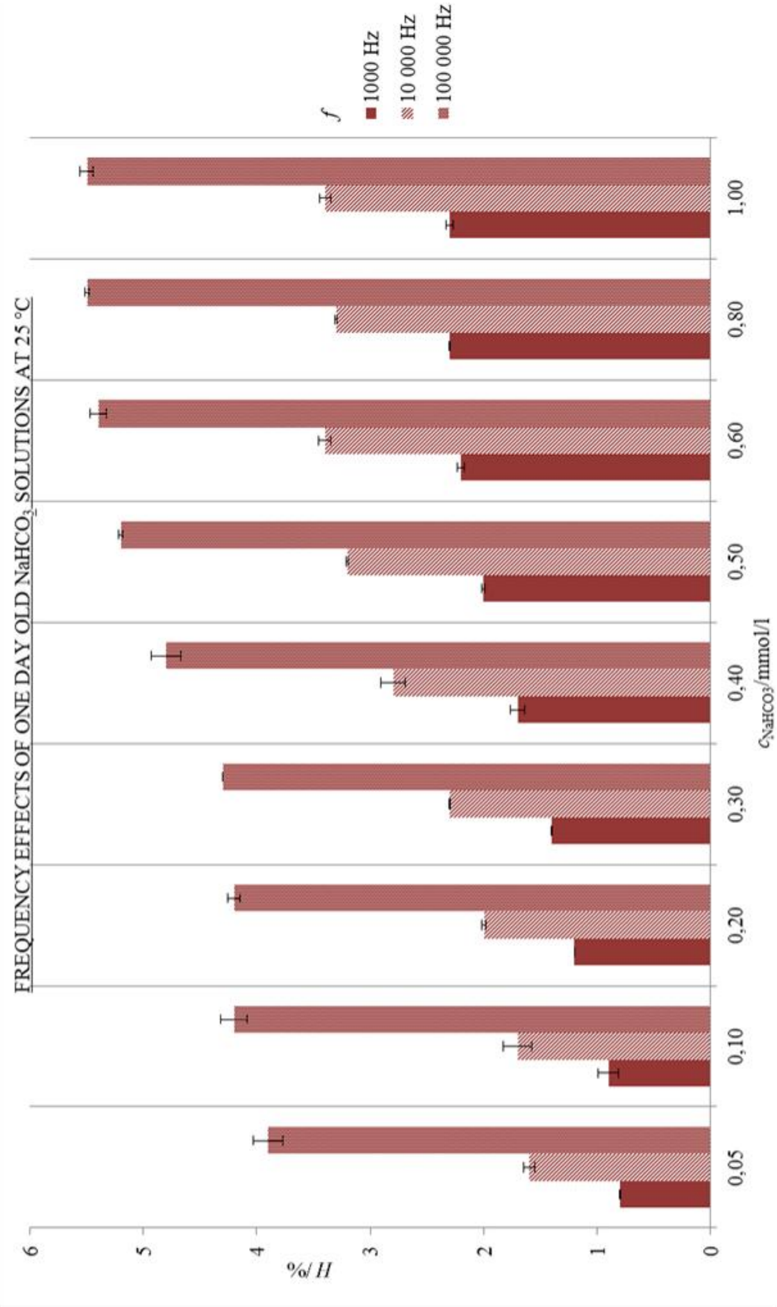

Figure S.6. Frequency effects with intervals of differences between duplicates at 1000 (filled), 10,000 (striped) and 100,000 Hz (dotted) of one day old $NaHCO_3$ solutions at 5 ℃, thermostat bath with 10 % NaCl aq.

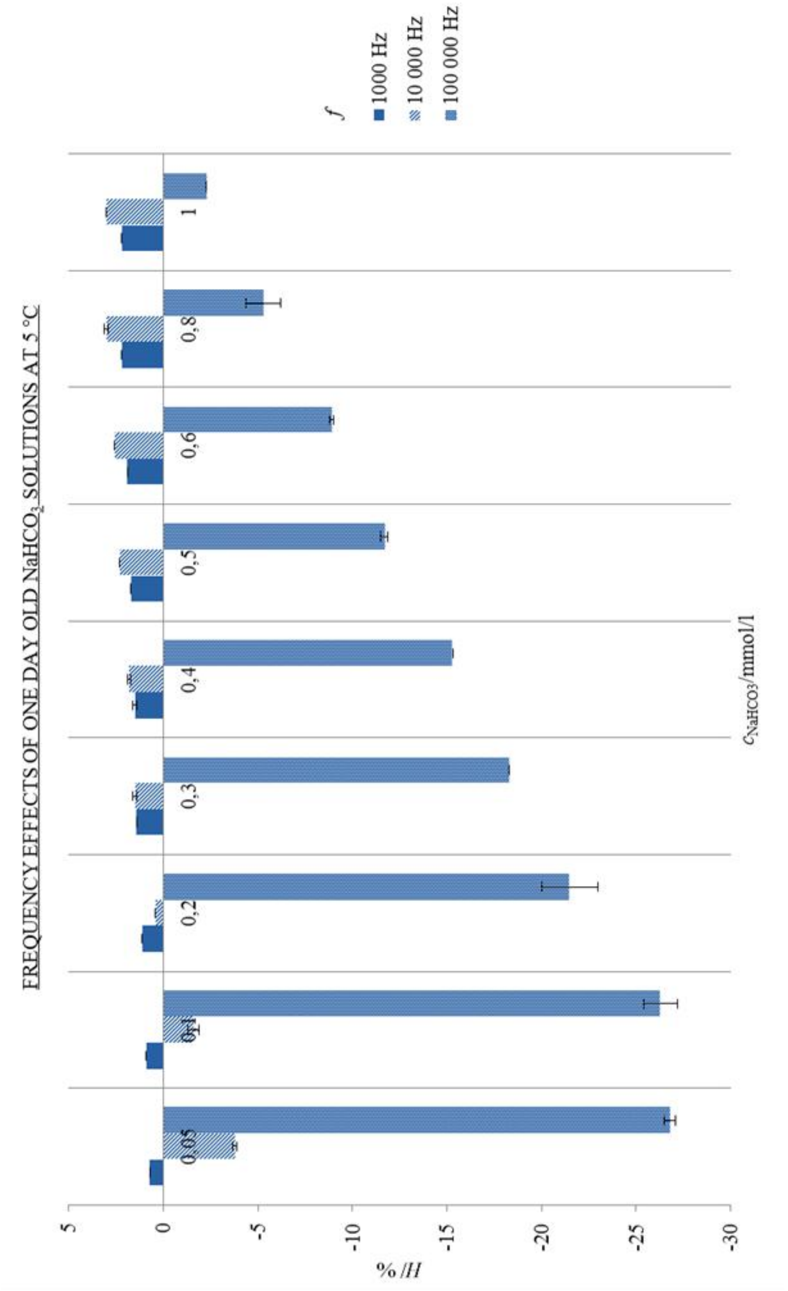

Table S.13. Frequency effects at 1000, 10,000 and 100,000 Hz of $NaHCO_3$ solutions at 25 in 5 ℃.

c_{NaHCO3}	*T* = 25 ℃			*T* = 5 ℃		
**(mmol/L)**	H_{1000} (%)	$H_{10,000}$ (%)	$H_{100,000}$ (%)	H_{1000} (%)	$H_{10,000}$ (%)	$H_{100,000}$ (%)
0.00	0.2	1.3	7.3	0.1	−11.8	−26.2
0.05	0.8	1.6	3.9	0.7	−3.8	−26.8
0.10	0.9	1.7	4.2	0.9	−1.6	−26.3
0.20	1.2	2.0	4.2	1.1	0.4	−21.5
0.30	1.4	2.3	4.3	1.4	1.5	−18.3

Table S.13. *Cont.*

c_{NaHCO3} (mmol/L)	*T* = 25 °C			*T* = 5 °C		
	H_{1000} (%)	$H_{10,000}$ (%)	$H_{100,000}$ (%)	H_{1000} (%)	$H_{10,000}$ (%)	$H_{100,000}$ (%)
0.40	1.7	2.8	4.8	1.5	1.8	−15.3
0.50	2	3.2	5.2	1.7	2.3	−11.7
0.60	2.2	3.4	5.4	1.9	2.6	−8.9
0.80	2.3	3.3	5.5	2.2	3.0	−5.3
1.00	2.3	3.4	5.5	2.2	3.0	−2.3

At 25 °C, when the conductivity was measured without a thermostat bath, the conductivity values slightly increased with increasing frequency, which is in agreement with Wachter and Barthel [3], while at 5 °C due to side electric currents the conductivity values in the concentration range 0 to 0.1 mmol/L $NaHCO_3$, at 10,000 and 100,000 Hz, are lower than at 120 Hz. The frequency effects at 1000 Hz are similar at both temperatures therefore the conductivities of aged solutions were compared at this frequency. At 25 °C the frequency effect is 2.3% and at 5 °C 2.2% (see Table S.13). Frequency effects at 10,000 Hz and 25 °C increased with increasing concentration from 1.3 to 3.4% and at 100,000 Hz from 4.0 to 7.3%. At 5 °C and 10,000 Hz, frequency effects in $NaHCO_3$ concentrations 0 to 0.1 mmol/L and in all used concentrations at 100,000 Hz had negative values.

2.1.7. Conductivity of One Day Old Treated Solutions

The conductivity of mechanically and electrically treated solutions was measured one day after treatment at 25 °C without a bath and at 5 °C with a thermostat bath, in 20-mL flasks (see Table S.14).

Table S.14. Conductivities of CON, MK and EK one day after the mechanic and electric treatment at 25 and 5 °C.

treatment	*T*	σ_{120}	σ_{1000}	$\sigma_{10,000}$	$\sigma_{100,000}$
	°C	µS/cm	µS/cm	µS/cm	µS/cm
CON	25	5.0	5.0	5.1	5.0
MK	25	5.0	5.0	5.0	5.1
EK	25	5.0	5.0	5.1	5.1
CON	5	3.1	3.1	2.3	2.0
MK	5	3.1	3.1	2.3	2.1
EK	5	3.2	3.2	2.4	2.2

The conductivity of the solutions at 25 and 5 °C treated mechanically and electrically one day after treatment does not differ from controls (see Table S.14 and Figure S.7). Therefore, we followed the findings of Elia's research group [2] and aged the solutions.

2.2. Testing the Working Hypothesis

2.2.1. Repeatability of Ageing

Table S.15. Repeatability of ageing—σ, H and c_{NaHCO_3} of the following treatments: untreated control solutions CON, mechanically treated MW and MK and electrically treated EW and EK of preparation PR1 (PR means aged exposed to light). PR1 aged for 310 days, σ measured at 25 ℃.

Treatment	Descriptive Statistics	σ_{1000}	c_{NaHCO3}	H_{1000}	$H_{10,000}$	$H_{100,000}$
		**µS/cm**	**mmol/L**	**%**	**%**	**%**
CON (5)	**AVG**	**46.5**	**0.19**	**2.5**	**4.0**	**6.2**
	SD	10.2	0.06	0.3	0.5	0.6
	SE	4.6	0.03	0.1	0.2	0.3
	RSE	9.8%	13.7%	5.8%	6.1%	4.3%
MW (7)	**AVG**	**43.0**	**0.20**	**2.6**	**4.0**	**6.2**
	SD	6.4	0.02	0.5	0.9	0.9
	SE	2.4	0.01	0.2	0.3	0.3
	RSE	5.6%	3.7%	7.9%	8.1%	5.6%
MK (7)	**AVG**	**40.7**	**0.18**	**2.1**	**3.3**	**5.4**
	SD	4.0	0.02	0.5	0.9	1.0
	SE	1.5	0.01	0.2	0.3	0.4
	RSE	3.7%	3.3%	9.5%	9.8%	6.8%
EW (5)	**AVG**	**44.0**	**0.20**	**1.9**	**3.0**	**5.4**
	SD	9.1	0.03	0.3	0.3	0.5
	SE	4.1	0.02	0.1	0.2	0.2
	RSE	9.2%	7.9%	5.8%	5.1%	3.9%
EK (5)	**AVG**	**43.7**	**0.20**	**1.9**	**2.9**	**5.1**
	SD	5.2	0.03	0.2	0.2	0.2
	SE	2.3	0.01	0.1	0.1	0.1
	RSE	5.3%	6.3%	3.8%	3.5%	2.2%
**PR1 (29)**	**AVG**	**43.3**	**0.19**	**2.2**	**3.5**	**5.7**
	SD	6.8	0.03	0.5	0.8	0.8
	SE	1.3	0.01	0.1	0.1	0.2
	RSE	2.9%	3.0%	4.0%	4.1%	2.7%

Table S.16. Repeatability of ageing—descriptive statistics with average (AVG), standard deviation (SD), standard error (SE) and relative standard error (RSE) values of σ_{1000}, c_{NaHCO3} and H of the following treatments: untreated control solutions CON, mechanically treated MW and MK and electrically treated EW and EK of preparation PR2 (PR means aged exposed to daylight). PR2 aged for 310 days, σ measured at 25 ℃.

Treatment	Descriptive Statistics	σ_{1000} µS/cm	c_{NaHCO3} mmol/L	H_{1000} %	$H_{10,000}$ %	$H_{100,000}$ %
**CON (5)**	**AVG**	**49.2**	**0.22**	**7.5**	**10.1**	**12.6**
	SD	11.7	0.03	0.8	1.0	1.2
	SE	5.2	0.01	0.4	0.5	0.5
	RSE	10.7%	6.4%	4.7%	4.4%	4.1%
**MW (5)**	**AVG**	**38.2**	**0.18**	**7.8**	**10.7**	**13.1**
	SD	8.7	0.03	1.0	1.4	1.5
	SE	3.9	0.01	0.5	0.6	0.7
	RSE	10.2%	6.3%	5.8%	5.7%	5.2%
**MK (7)**	**AVG**	**38.5**	**0.17**	**8.1**	**10.9**	**13.6**
	SD	10.2	0.03	1.2	1.7	1.9
	SE	3.8	0.01	0.5	0.6	0.7
	RSE	10.0%	5.7%	5.8%	5.8%	5.2%
**EW (5)**	**AVG**	**48.2**	**0.21**	**8.5**	**11.5**	**14.0**
	SD	7.6	0.03	0.5	0.6	0.8
	SE	3.4	0.01	0.2	0.3	0.4
	RSE	7.1%	6.2%	2.6%	2.4%	2.5%
**EK (5)**	**AVG**	**45.3**	**0.19**	**8.1**	**11.0**	**13.3**
	SD	2.4	0.01	0.2	0.2	0.3
	SE	1.1	0.01	0.1	0.1	0.1
	RSE	2.3%	2.9%	1.3%	0.8%	1.0%
**PR2 (27)**	**AVG**	**43.5**	**0.19**	**8.0**	**10.9**	**13.6**
	**SD**	**9.5**	**0.03**	**0.9**	**1.2**	**1.3**
	**SE**	**1.8**	**0.01**	**0.2**	**0.2**	**0.2**
	**RSE**	**4.2%**	**2.9%**	**2.1%**	**2.1%**	**1.8%**

Table S.17. Repeatability of conductivity on ageing of preparations 1 and 2 (PR1 and PR2) at 25 ℃ and 1000 Hz, if treatments were combined. Descriptive statistics of σ the 1st and 310th day with AVG, N, SE and RSE.

Ageing [d]	Descriptive Statistics	PR1	PR2
1	**AVG σ/µS/cm**	**12.5**	
	N/	56	
	SE/µS/cm	0.19	
	RSE/%	1.5	
310	**AVG σ/µS/cm**	**43.3**	**43.5**
	N/	29	27
	SE/µS/cm	1.3	1.8
	RSE/%	2.9	4.2

Table S.18. Repeatability of σ at 1000 Hz on ageing. Descriptive statistics of PR1 and PR2 treatments—σ with AVG, SD, N, SE and RSE and normality tests Shapiro-Wilk (TNP S-W).

Treatment	Descriptive Statistics	PR1	PR2
CON	**AVG σ/µS/cm**	**46.5**	**49.2**
	SD/µS/cm	10.2	11.7
	N/	5	5
	SE/µS/cm	4.58	5.24
	RSE/%	9.8	10.6
	TNP S-W_{CON} (Sig.)	0.604	
MW	**AVG σ/µS/cm**	**43.0**	**38.2**
	SD/µS/cm	6.42	8.69
	N/	7	5
	SE/µS/cm	2.43	3.89
	RSE/%	5.6	10.2
	TNP S-W_{MW} (Sig.)	0.579	
MK	**AVG σ/µS/cm**	**40.7**	**48.5**
	SD/µS/cm	4.03	1.02
	N/	7	7
	SE/µS/cm	1.52	3.84
	RSE/%	3.7	10.0
	TNP S-W_{MK} (Sig.)	0.021	
EW	**AVG σ/µS/cm**	**44.0**	**48.2**
	SD/µS/cm	9.11	7.62
	N/	5	5
	SE/µS/cm	4.07	3.41
	RSE/%	9.3	7.1
	TNP S-W_{EW} (Sig.)	0.042	
EK	**AVG σ/µS/cm**	**43.7**	**45.3**
	SD/µS/cm	5.14	2.39
	N/	5	5
	SE/µS/cm	2.30	1.07
	RSE/%	5.3	2.4
	TNP S-W_{EK} (Sig.)	0.022	

Table S.19. Comparison of PR1 and PR2 on treatment by univariate analysis, UA, (dependent variable: σ_{1000}; fixed factor: preparation, treatment) and linear mixed model, LMM, (subject: flask; dependent variable: σ_{1000}; factor: preparation, treatment; full factorial analysis).

Interactions	UA	LMM
preparation*treatment	1.000	0.664
preparation	1.000	0.887
treatment	0.078	0.078

Table S.20. Influence of ageing on frequency effects (*H*) of preparations PR1 and PR2, if treatments were combined. Descriptive statistics of *H* the 1st and 310th day with AVG, N and SE.

Ageing [d]	Descriptive Statistics	PR1			PR2		
		**1000 Hz**	**10,000 Hz**	**100,000 Hz**	**1000 Hz**	**10,000 Hz**	**100,000 Hz**
1	**AVG *H*/%**	**0.8**	**1.6**	**3.9**	**0.9**	**1.7**	**4.2**
	N/	29	29	29	27	27	27
	SE/%	0.0	0.0	0.6	0.1	0.1	0.1
310	**AVG *H*/%**	**2.2**	**3.5**	**5.7**	**8.0**	**10.8**	**13.5**
	N/	29	29	29	27	27	27
	SE/%	0.1	0.1	0.2	0.2	0.2	0.2

2.2.2. Influence of Ageing Volume

Table S.21. Influence of ageing *V*—descriptive statistics with average (AVG), standard deviation (SD), standard error (SE) and relative standard error (RSE) values of σ_{1000}, σ/σ_{CC1000}, c_{NaHCO3} and *H*. Ageing for 370 days in 10, 5 and 2 mL of 20-mL flasks. σ measured at 25 ℃ in 2.5-mL flasks.

Volume	Descriptive Statistics	σ_{1000}	σ/σ_{CC1000}	c_{NaHCO3}	H_{1000}	$H_{10,000}$	$H_{100,000}$
		**µS/cm**	**%**	**mmol/L**	**%**	**%**	**%**
**10 mL (10)**	**AVG**	**30.8**	**136.8**	**0.17**	**4.4**	**6.2**	**8.4**
	SD	3.8	6.9	0.02	0.4	0.5	0.7
	SE	1.2	2.2	0.01	0.1	0.2	0.2
	RSE	3.9%	1.6%	4.4%	2.5%	2.5%	2.7%
**5 mL (10)**	**AVG**	**51.5**	**156.7**	**0.28**	**6.3**	**8.7**	**10.8**
	SD	3.8	6.0	0.02	0.4	0.4	0.8
	SE	1.2	1.9	0.01	0.1	0.1	0.3
	RSE	2.3%	1.2%	2.8%	2.0%	1.6%	2.4%
**2 mL (8)**	**AVG**	**106.2**	**176.3**	**0.58**	**10.0**	**13.4**	**16.3**
	SD	2.9	2.2	0.02	0.3	0.3	0.3
	SE	1.0	0.8	0.01	0.1	0.1	0.1
	RSE	1.0%	0.4%	1.2%	0.9%	0.7%	0.6%

Table S.22. Influence of ageing *V* on conductivity σ at 25 ℃ and 1000 Hz. Descriptive statistics of 2, 5 and 10 mL σ the 1st and 370th day with AVG, N, SE and RSE.

Ageing [d]	Descriptive Statistics	2 mL	5 mL	10 mL
1	**AVG σ/µS/cm**		**12.5**	
	N/		28	
	SE/ µS/cm		0.2	
	RSE/%		1.5	
370	**AVG σ/µS/cm**	**106.0**	**51.5**	**30.8**
	N/	8	10	10
	SE/ µS/cm	1.0	1.2	1.2
	RSE/%	1.0	2.3	3.9

Table S.23. Influence of ageing *V* on σ/σ_{CC} at 1000 Hz upon ageing. σ^E/σ and descriptive statistics of 2 (4.0 cm^{-1}), 5 (2.6 cm^{-1}) and 10 mL (2.1 cm^{-1}) σ/σ_{CC} with AVG, SD, N, SE and RSE.

descriptive statistics	4.0 cm^{-1}	2.6 cm^{-1}	2.1 cm^{-1}
**AVG σ/σ_{CC}/%**	**176.3**	**156.7**	**136.8**
SD/%	2.2	6.0	6.9
N/	8	10	10
SE/%	0.8	1.9	2.2
RSE/%	0.4	1.2	1.6
**AVG σ^E/σ/%**	**43.3**	**36.1**	**26.8**

Table S.24. Influence of ageing *V* on frequency effects (*H*). Descriptive statistics of 2, 5 and 10 mL *H* the 1st and 370th day with AVG, N and SE.

Ageing [d]	Descriptive Statistics	2 mL			5 mL			10 mL		
		1000 Hz	10,000 Hz	100,000 Hz	1000 Hz	10,000 Hz	100,000 Hz	1000 Hz	10,000 Hz	100,000 Hz
1	**AVG *H*/%**	**0.8**	**1.6**	**3.9**	**0.8**	**1.6**	**3.9**	**0.8**	**1.6**	**3.9**
	N/	8	8	8	10	10	10	10	10	10
	SE/%	0.0	0.0	0.6	0.0	0.0	0.6	0.0	0.0	0.6
370	**AVG *H*/%**	**10.0**	**13.4**	**16.3**	**6.3**	**8.7**	**10.8**	**4.4**	**6.2**	**8.4**
	N/	8	8	8	10	10	10	10	10	10
	SE/%	0.1	0.1	0.1	0.1	0.1	0.3	0.1	0.2	0.2

Table S.25. Influence of ageing *V* on frequency effects of aged and fresh solutions with equal concentrations of $NaHCO_3$, if treatments were combined.

V/mL	One Day Old *H* [%]			370 Days Old *H* [%]		
	1000	10,000	100,000	1000	10,000	100,000
10	1.2	2.0	4.2	4.4	6.2	8.4
5	1.4	2.3	4.3	6.3	8.7	10.8
2	2.2	3.4	5.4	10	13.4	16.3

2.2.3. Influence of Ageing Condition, Treatment and Temperature

Table S.26. Influence of ageing condition and treatment—descriptive statistics with average (AVG), standard deviation (SD), standard error (SE) and relative standard error (RSE) values of σ_{1000}, c_{NaHCO3}, σ/σ_{CC1000} and H. The following treatments: untreated control solutions CON, mechanically treated MW and MK and electrically treated EW and EK were aged for 310 days under conditions PR—exposed to daylight, ST—protected from daylight and MD—at −20 °C. σ measured at 25 °C.

Condition	Treatment	Descriptive Statistics	σ_{1000} µS/cm	σ/σ_{CC1000} %	c_{NaHCO3} mmol/L	H_{1000} %	$H_{10,000}$ %	$H_{100,000}$ %
PR	CON (5)	**AVG**	**46.5**	**175.0**	**0.19**	**2.5**	**4.0**	**6.2**
		SD	10.2	18.7	0.06	0.3	0.5	0.6
		SE	4.6	8.4	0.03	0.1	0.2	0.3
		RSE	9.8%	4.8%	13.7%	5.8%	6.1%	4.3%
	MW (6)	**AVG**	**42.9**	**168.5**	**0.20**	**2.5**	**3.8**	**6.1**
		SD	7.0	16.2	0.02	0.5	0.8	0.9
		SE	2.9	6.6	0.01	0.2	0.3	0.4
		RSE	6.7%	3.9%	4.1%	8.8%	9.0%	6.2%
	MK (7)	**AVG**	**40.7**	**170.7**	**0.18**	**2.1**	**3.3**	**5.4**
		SD	4.0	9.0	0.02	0.5	0.9	1.0
		SE	1.5	3.4	0.01	0.2	0.3	0.4
		RSE	3.7%	2.0%	3.3%	9.5%	9.8%	6.8%
	EW (5)	**AVG**	**44.0**	**173.1**	**0.20**	**1.9**	**3.0**	**5.4**
		SD	9.1	13.0	0.03	0.3	0.3	0.5
		SE	4.1	5.8	0.02	0.1	0.2	0.2
		RSE	9.3%	3.4%	7.9%	5.8%	5.1%	3.9%
	EK (5)	**AVG**	**43.7**	**173.4**	**0.20**	**1.9**	**2.9**	**5.1**
		SD	5.1	8.7	0.03	0.2	0.2	0.2
		SE	2.3	3.9	0.01	0.1	0.1	0.1
		RSE	5.3%	2.2%	6.3%	3.8%	3.5%	2.2%
	**PR (28)**	**AVG**	**43.3**	**171.9**	**0.19**	**2.2**	**3.4**	**5.6**
		SD	6.9	12.7	0.03	0.5	0.7	0.8
		SE	1.3	2.4	0.01	0.1	0.1	0.2
		RSE	3.0%	1.4%	3.1%	4.0%	4.0%	2.7%
ST	CON (3)	**AVG**	**58.3**	**187.7**	**0.26**	**3.3**	**4.9**	**7.0**
		SD	3.1	10.3	0.03	0.7	1.1	1.4
		SE	1.8	6.0	0.02	0.4	0.7	0.8
		RSE	3.1%	3.2%	7.0%	12.8%	13.3%	11.5%
	MW (4)	**AVG**	**57.6**	**192.0**	**0.25**	**2.6**	**3.9**	**6.0**
		SD	14.3	9.7	0.08	0.4	0.5	0.6
		SE	7.1	4.8	0.04	0.2	0.2	0.3
		RSE	12.4%	2.5%	15.7%	7.5%	6.2%	4.9%

Table S.26. *Cont.*

Condition	Treatment	Descriptive Statistics	σ_{1000} µS/cm	σ/σ_{CC1000} %	c_{NaHCO3} mmol/L	H_{1000} %	$H_{10,000}$ %	$H_{100,000}$ %
ST	MK (4)	**AVG**	**40.0**	**159.3**	**0.20**	**2.2**	**3.3**	**5.3**
		SD	8.7	17.2	0.05	0.5	0.7	0.6
		SE	4.4	8.6	0.02	0.3	0.4	0.3
		RSE	10.9%	5.4%	11.9%	12.1%	11.0%	6.1%
	EW (5)	**AVG**	**43.0**	**179.8**	**0.18**	**2.7**	**4.0**	**6.4**
		SD	7.7	26.2	0.02	0.4	0.7	0.7
		SE	3.5	11.7	0.01	0.2	0.3	0.3
		RSE	8.1%	6.5%	5.6%	7.1%	7.7%	4.9%
	EK (4)	**AVG**	**43.1**	**167.4**	**0.20**	**2.6**	**4.0**	**6.2**
		SD	8.9	18.5	0.03	0.2	0.3	0.4
		SE	4.4	9.3	0.01	0.1	0.1	0.2
		RSE	10.3%	5.5%	7.6%	3.9%	3.7%	2.8%
	**ST (20)**	**AVG**	**47.6**	**176.8**	**0.21**	**2.6**	**4.0**	**6.1**
		SD	11.5	20.6	0.05	0.5	0.8	0.9
		SE	2.6	4.6	0.01	0.1	0.2	0.2
		RSE	5.4%	2.6%	5.5%	4.5%	4.4%	3.2%
MD	CON (3)	**AVG**	**11.2**	**87.5**	**0.06**	**1.0**	**2.0**	**5.1**
		SD	0.6	3.3	0.01	0.0	0.2	0.6
		SE	0.4	1.9	0.00	0.0	0.1	0.3
		RSE	3.2%	2.1%	5.3%	0.0%	5.0%	6.5%
	MW (3)	**AVG**	**12.3**	**98.2**	**0.06**	**1.1**	**2.0**	**4.7**
		SD	0.9	6.5	0.00	0.1	0.2	0.1
		SE	0.5	3.7	0.00	0.1	0.1	0.1
		RSE	4.1%	3.8%	0.0%	5.2%	4.3%	1.2%
	MK (5)	**AVG**	**10.2**	**82.8**	**0.06**	**0.9**	**1.8**	**4.8**
		SD	0.6	3.1	0.00	0.1	0.1	0.2
		SE	0.3	1.4	0.00	0.0	0.1	0.1
		RSE	2.5%	1.7%	3.4%	4.3%	3.0%	1.7%
	EW (4)	**AVG**	**10.7**	**82.9**	**0.07**	**1.0**	**1.9**	**4.9**
		SD	0.7	4.0	0.01	0.0	0.0	0.1
		SE	0.4	2.0	0.00	0.0	0.0	0.1
		RSE	3.3%	2.4%	4.4%	0.0%	1.3%	1.0%
	EK (5)	**AVG**	**10.5**	**82.1**	**0.06**	**1.0**	**1.9**	**5.4**
		SD	0.6	3.6	0.00	0.1	0.2	0.9
		SE	0.3	1.6	0.00	0.0	0.1	0.4
		RSE	2.6%	2.0%	3.2%	5.0%	4.8%	7.1%
	**MD (20)**	**AVG**	**10.8**	**85.6**	**0.06**	**1.0**	**1.9**	**5.0**
		SD	0.9	6.7	0.00	0.1	0.2	0.5
		SE	0.2	1.5	0.00	0.0	0.0	0.1
		RSE	1.9%	1.8%	1.8%	2.0%	1.9%	2.4%

Table S.27. Influence of ageing condition and treatment—descriptive statistics with average (AVG), standard deviation (SD), standard error (SE) and relative standard error (RSE) values of σ_{1000}, c_{NaHCO3}, σ/σ_{CC1000} and H. The following treatments: untreated control solutions CON, mechanically treated MW and MK and electrically treated EW and EK were aged for 310 days under conditions PR—exposed to daylight, ST—protected from daylight and MD—at −20 ℃. σ measured at 5 ℃.

Condition	Treatment	Descriptive Statistics	σ_{1000}	σ/σ_{CC1000}	c_{NaHCO3}	H_{1000}	$H_{10,000}$	$H_{100,000}$
			µS/cm	%	mmol/L	%	%	%
PR	CON (5)	**AVG**	**28.7**	**166.4**	**0.19**	**1.6**	**2.0**	**−15.6**
		SD	6.0	18.9	0.06	0.2	0.4	3.6
		SE	2.7	8.4	0.03	0.1	0.2	1.6
		RSE	9.4%	5.1%	13.7%	6.1%	8.0%	−10.3%
	MW (6)	**AVG**	**26.9**	**161.9**	**0.20**	**2.2**	**2.6**	**−15.2**
		SD	4.1	14.0	0.02	1.4	1.9	2.5
		SE	1.7	5.7	0.01	0.6	0.8	1.0
		RSE	6.2%	3.5%	4.1%	26.5%	29.3%	−6.7%
	MK (7)	**AVG**	**24.2**	**155.2**	**0.18**	**1.8**	**2.2**	**−17.1**
		SD	2.6	9.2	0.02	1.3	1.8	2.1
		SE	1.0	3.5	0.01	0.5	0.7	0.8
		RSE	4.0%	2.3%	3.3%	25.9%	30.9%	−4.7%
	EW (5)	**AVG**	**26.0**	**157.2**	**0.20**	**1.5**	**1.7**	**−17.0**
		SD	4.5	7.7	0.03	0.3	0.5	3.4
		SE	2.0	3.4	0.02	0.1	0.2	1.5
		RSE	7.7%	2.2%	7.9%	8.8%	12.3%	−8.8%
	EK (5)	**AVG**	**28.6**	**173.8**	**0.20**	**1.4**	**1.6**	**−15.9**
		SD	3.9	7.3	0.03	0.2	0.4	1.8
		SE	1.7	3.3	0.01	0.1	0.2	0.8
		RSE	6.1%	1.9%	6.3%	7.0%	10.3%	−5.2%
	**PR (28)**	**AVG**	**26.7**	**162.3**	**0.19**	**1.7**	**2.1**	**−16.2**
		SD	4.3	13.1	0.03	0.9	1.2	2.6
		SE	0.8	2.5	0.01	0.2	0.2	0.5
		RSE	3.0%	1.5%	3.1%	10.0%	11.4%	−3.1%
ST	CON (3)	**AVG**	**35.9**	**178.1**	**0.26**	**2.3**	**3.2**	**−8.2**
		SD	3.4	13.9	0.03	0.6	1.0	2.9
		SE	1.9	8.1	0.02	0.3	0.6	1.7
		RSE	5.4%	4.5%	7.0%	14.3%	17.4%	−20.6%
	MW (4)	**AVG**	**36.8**	**188.3**	**0.25**	**1.7**	**2.4**	**−10.4**
		SD	10.2	10.4	0.08	0.3	0.6	4.3
		SE	5.1	5.2	0.04	0.2	0.3	2.1
		RSE	13.8%	2.8%	15.7%	9.9%	12.7%	−20.7%
	MK (4)	**AVG**	**27.4**	**167.0**	**0.20**	**1.5**	**1.8**	**−15.5**
		SD	5.8	11.8	0.05	0.3	0.6	4.0
		SE	2.9	5.9	0.02	0.1	0.3	2.0
		RSE	10.5%	3.5%	11.9%	8.5%	17.1%	−13.0%

Table S.27. *Cont.*

Condition	Treatment	Descriptive Statistics	σ_{1000} µS/cm	σ/σ_{CC1000} %	c_{NaHCO3} mmol/L	H_{1000} %	$H_{10,000}$ %	$H_{100,000}$ %
ST	EW (5)	**AVG**	**28.2**	**180.7**	**0.18**	**1.5**	**2.0**	**−14.5**
		SD	2.3	3.2	0.02	0.2	0.3	1.6
		SE	1.0	1.4	0.01	0.1	0.2	0.7
		RSE	3.7%	0.8%	5.6%	4.7%	7.6%	−4.8%
	EK (4)	**AVG**	**28.7**	**171.5**	**0.20**	**1.9**	**2.4**	**−13.6**
		SD	4.5	11.1	0.03	0.6	0.7	1.9
		SE	2.2	5.6	0.01	0.3	0.3	0.9
		RSE	7.8%	3.2%	7.6%	15.7%	13.7%	−6.9%
	**ST (20)**	**AVG**	**31.0**	**177.3**	**0.21**	**1.7**	**2.3**	**−12.7**
		SD	6.6	11.8	0.05	0.5	0.7	3.8
		SE	1.5	2.6	0.01	0.1	0.2	0.8
		RSE	4.8%	1.5%	5.5%	5.9%	7.0%	−6.7%
MD	CON (3)	**AVG**	**7.5**	**86.4**	**0.06**	**0.7**	**−4.4**	**−26.1**
		SD	0.5	4.5	0.01	0.1	0.9	1.6
		SE	0.3	2.6	0.00	0.0	0.5	0.9
		RSE	4.2%	3.0%	5.3%	5.0%	−12.0%	−3.4%
	MW (3)	**AVG**	**8.5**	**100.4**	**0.06**	**1.4**	**−2.2**	**−26.7**
		SD	2.0	21.6	0.00	0.2	2.1	5.3
		SE	1.1	12.5	0.00	0.1	1.2	3.0
		RSE	13.3%	12.4%	0.0%	8.4%	−53.1%	−11.4%
	MK (5)	**AVG**	**7.2**	**86.4**	**0.06**	**0.4**	**−5.2**	**−25.6**
		SD	1.0	10.8	0.00	0.1	1.0	2.7
		SE	0.4	4.8	0.00	0.0	0.4	1.2
		RSE	5.9%	5.6%	3.4%	12.9%	−8.6%	−4.8%
	EW (4)	**AVG**	**6.8**	**77.9**	**0.07**	**0.7**	**−5.0**	**−27.4**
		SD	0.7	8.7	0.01	0.3	0.6	2.4
		SE	0.4	4.3	0.00	0.2	0.3	1.2
		RSE	5.5%	5.6%	4.4%	22.1%	−6.1%	−4.5%
	EK (5)	**AVG**	**7.0**	**81.7**	**0.06**	**0.7**	**−4.3**	**−25.3**
		SD	0.4	6.5	0.00	0.4	0.4	1.9
		SE	0.2	2.9	0.00	0.2	0.2	0.8
		RSE	2.5%	3.5%	3.2%	22.1%	−4.5%	−3.3%
	**MD (20)**	**AVG**	**7.3**	**85.6**	**0.06**	**0.7**	**−4.4**	**−26.1**
		SD	1.0	12.2	0.00	0.4	1.4	2.7
		SE	0.2	2.7	0.00	0.1	0.3	0.6
		RSE	3.1%	3.2%	1.8%	12.2%	−6.9%	−2.3%

No statistically significant differences were found between the conductivities of treatments CON, MW, MK, EW and EK measured at 25 and 5 ℃. Therefore treatments were combined in Table S.28.

Table S.28. Influence of ageing condition—descriptive statistics with average (AVG), standard deviation (SD), standard error (SE) and relative standard error (RSE) values of σ_{1000}, c_{NaHCO3}, σ/σ_{CC1000} and *H*. The solutions were aged for 310 days under conditions PR—exposed to daylight, ST—protected from daylight and MD—at −20 ℃. Treatments are combined. σ was measured at 25 and 5 ℃.

T ℃	Treatment	Descriptive Statistics	σ_{1000} µS/cm	σ/σ_{CC1000} %	c_{NaHCO3} mmol/L	H_{1000} %	$H_{10,000}$ %	$H_{100,000}$ %
25	PR (28)	**AVG**	**43.3**	**171.9**	**0.19**	**2.2**	**3.4**	**5.6**
		SD	6.9	12.7	0.03	0.5	0.7	0.8
		SE	1.3	2.4	0.0	0.1	0.1	0.2
		RSE	3.0%	1.4%	3.1%	4.0%	4.0%	2.7%
	ST (20)	**AVG**	**47.6**	**176.8**	**0.21**	**2.6**	**4.0**	**6.1**
		SD	11.5	20.6	0.05	0.5	0.8	0.9
		SE	2.6	4.6	0.0	0.1	0.2	0.2
		RSE	5.4%	2.6%	5.5%	4.5%	4.4%	3.2%
	MD (20)	**AVG**	**10.8**	**85.6**	**0.06**	**1.0**	**1.9**	**5.0**
		SD	0.9	6.7	0.00	0.1	0.2	0.5
		SE	0.2	1.5	0.0	0.0	0.0	0.1
		RSE	1.9%	1.8%	1.8%	2.0%	1.9%	2.4%
5	PR (28)	**AVG**	**26.7**	**162.3**	**0.19**	**1.7**	**2.1**	**−16.2**
		SD	4.3	13.1	0.03	0.9	1.2	2.6
		SE	0.8	2.5	0.0	0.2	0.2	0.5
		RSE	3.0%	1.5%	3.1%	10.0%	11.4%	−3.1%
	ST (20)	**AVG**	**31.0**	**177.3**	**0.21**	**1.7**	**2.3**	**−12.7**
		SD	6.6	11.8	0.05	0.5	0.7	3.8
		SE	1.5	2.6	0.0	0.1	0.2	0.8
		RSE	4.8%	1.5%	5.5%	5.9%	7.0%	−6.7%
	MD (20)	**AVG**	**7.3**	**85.6**	**0.06**	**0.7**	**−4.4**	**−26.1**
		SD	1.0	12.2	0.00	0.4	1.4	2.7
		SE	0.2	2.7	0.0	0.1	0.3	0.6
		RSE	3.1%	3.2%	1.8%	12.2%	−6.9%	−2.3%

Table S.29. Influence of ageing condition on conductivity at 1000 Hz measured at 5 ℃ and 25 ℃, if treatments were combined. Descriptive statistics of conditions PR, ST and MD the 1st and 310th day of ageing with AVG, N, SE and RSE.

Ageing [d]	Descriptive Statistics	T = 25 ℃			T = 5 ℃		
		PR	ST	MD	PR	ST	MD
1	**AVG σ/µS/cm**		**9.0**			**7.2**	
	N/		68			68	
	SE/µS/cm		0.2			0.0	
	RSE/%		1.5			0.5	
310	**AVG σ/µS/cm**	**43.3**	**47.6**	**10.8**	**26.7**	**31.0**	**7.3**
	N/	28	20	20	28	20	20
	SE/µS/cm	1.3	2.6	0.2	0.8	1.5	0.2
	RSE/%	3.0	5.4	1.9	3.1	4.8	3.1

Table S.30. Influence of temperature on σ/σ_{CC} if conditions, frequencies and treatments were combined.

descriptive statistics	25 °C	5 °C
**AVG σ/σ_{CC}/%**	**144.1**	**147.1**
SE/%	1.7	1.5

Table S.31. Influence of ageing conditions on frequency effects, if treatments CON, MW, MK, EW and EK were combined. Descriptive statistics of PR, ST and MD H the 1st and 370th day with AVG, N and SE at 25 °C.

Descriptive Statistics	PR			ST			MD		
	1000	10,000	100,000	1000	10,000	100,000	1000	10,000	100,000
**AVG $H_{1.\ day}$/%**	**0.8**	**1.6**	**3.9**	**0.8**	**1.6**	**3.9**	**0.8**	**1.6**	**3.9**
N/	28	28	28	20	20	20	20	20	20
SE/%	0.0	0.0	0.6	0.0	0.0	0.6	0.0	0.0	0.6
**AVG $H_{310.\ day}$/%**	**2.2**	**3.5**	**5.7**	**2.6**	**4.0**	**6.1**	**1.0**	**1.9**	**5.0**
N/	28	28	28	20	20	20	20	20	20
SE/%	0.1	0.1	0.2	0.1	0.2	0.2	0.0	0.0	0.1

References

1. ICUMSA record of 1966. Available online: http://eur-lex.europa.eu/LexUriServ/LexUriServ.do?uri=CELEX:31969R1265:SL:HTML (accessed on 4 December 2011).
2. Elia, V.; Elia, L.; Montanino, M.; Napoli, E.; Niccoli, M.; Nonatelli, L. Conductometric studies of the serially diluted and agitated solutions on an anomalous effect that depends on the dilution process. *J. Mol. Liq.* **2007**, *135*, 158–165.
3. Wachter, R.; Barthel, J. Untersuchungen zur temperaturabhängigkeit der eigenschaften von elektrolytlösungen II. Bestimmung der leitfähigkeit über einen groβen temperaturbereich. *Ber. Bunsenges. Phys. Chem.* **1979**, *83*, 634–642.

© 2012 by the authors; licensee MDPI, Basel, Switzerland. This article is an open access article distributed under the terms and conditions of the Creative Commons Attribution license (http://creativecommons.org/licenses/by/3.0/).
